# Flammability of Thick but Thermally Thin Materials including Bio-Based Materials

**DOI:** 10.3390/molecules28135175

**Published:** 2023-07-03

**Authors:** Rodolphe Sonnier, Loïc Dumazert, Arnaud Regazzi, Lily Deborde, Christophe Lanos

**Affiliations:** 1 Polymers Composites and Hybrids (PCH), IMT Mines Ales, 30319 Ales, France; loic.dumazert@mines-ales.fr; 2 LMGC, IMT Mines Ales, University Montpellier, CNRS, 30100 Ales, France; arnaud.regazzi@mines-ales.fr; 3 Laboratoire de Génie Civil et Génie Mécanique, University Rennes, 3 rue du Clos Courtel, 35704 Rennes, France; lily.deborde@univ-rennes1.fr (L.D.); christophe.lanos@univ-rennes1.fr (C.L.)

**Keywords:** insulating materials, cone calorimeter, thermally thin materials, fire behavior, bio-resources, hemp fibers, wood

## Abstract

The fire reaction of various types of flammable lightweight materials is investigated using a cone calorimeter. The influences of parameters such as sample density, sample mass, effective heat of combustion and heat flux on the mass loss after exposition are discussed. Interpretations of the hemp fibers’ tests results lead us to propose a phenomenological model able to calculate the peak of heat release rate (pHRR) of such thermally thin materials, with or without flame retardant. A database gathering the whole results of tests performed on a large set of materials including fibers, bio-resources panels, bio-based concretes and fabrics is used to validate the proposed model. Interestingly, the model is found to be relevant also for denser wood specimens. The model is based on the distinction of the contributions of the exposed top layer and the deeper layer to the combustion. Indeed, in such materials, the heat conduction is limited (either by the intrinsic properties of the material or by the formation of an insulating char) and therefore the pHRR only depends on a limited volume of materials directly absorbing the heat flux from the radiant cone. Accuracy and limitations of the model are discussed.

## 1. Introduction

The use of biobased materials such as plant fibers as part of the formulation of materials is highly exploited by various industry sectors. Indeed, in the case of building materials, the use of agricultural products represents an attractive alternative for several reasons [1]:

- plants sequester CO_2_ during their growth, providing a resource with a positive carbon balance;
- agro-resources present high intrinsic technical performances: thermal and acoustic insulation, water vapor regulation, good mechanical properties;
- their valorization contributes to the development of territories and favors the development of activities in short circuits.

Hemp is a good example of such annually renewable plant resource. The entire plant can be valorized, and integrating hemp into the cultivation rotation has agricultural advantages [2]. Hemp fibers, wood fibers, flax fibers, recycled cotton, cellulose wadding and sheep’s wool are already used to produce good thermal insulation, with high hygroscopic and acoustic properties [3,4,5]. As an example, bulk hemp fibers present a thermal conductivity lower than 0.045 W/(m.K) for a bulk density around 28 kg/m^3^. The same fibers, bound to form a semi-rigid thermal insulation panel lead to a thermal conductivity less than 0.038 W/(m.K) for a density around 35 kg/m^3^. Such performances are close to the ones obtained with other fibrous thermal insulation materials such as glass wool and rock wool. However, as a cellulosic material, the bio-resources are vulnerable to fire [6].

In order to predict the fire behavior of a material, a fundamental aspect is to consider its thermal thickness (called the depth of thermal penetration). Materials can be considered thermally thin or thermally thick depending on their thermal behavior. Especially in cone calorimeter, both behaviors lead to different heat release rate (HRR) curves [7].

There are several ways to understand what a thermally thin or thick behavior means. When a thermally thick material is heated, heat transfer from the surface to the bulk is rapid (high thermal diffusivity called a) and leads to a slow increase in the material temperature. A heat gradient is established through its thickness. This behavior usually delays the ignition (even if a strong heat transfer to the bulk can also lead to a high peak of heat release rate (pHRR) reaching at a later stage [8]). In that case, time-to-ignition (TTI) is proportional to the reciprocal of the squared heat flux, according to Quintiere’s model [9,10]. For thermally thin materials, on the contrary, thermal diffusivity is not high enough to transfer heat rapidly from the surface to the bulk. The surface material temperature increase is rapid. Time-to-ignition no longer depends on thermal diffusivity and remains proportional to the reciprocal of the heat flux [7].

Moreover, it is often considered that there is no heat gradient through the thickness of thermally thin materials. Nevertheless, this definition corresponds only to thin materials such as fabrics or films. It is too restrictive because some thick materials have a thermally thin behavior despite a high thickness. This is especially the case with thick insulating materials such as foams, for example. In these materials, the temperature is actually obviously not homogeneous through the thickness: the top surface is heated very quickly while the bulk is heated only after some time (by conductive heat transfer or by radiative transfer after ablation of the top surface). Such materials burn progressively, and the period of burning depends on their thickness. But the instantaneous response to external heat flux corresponds to a thermally thin behavior: a finite top volume is heated, and heat transfer to the bulk is negligible. Therefore, TTI is proportional to the reciprocal of the heat flux.

The thermal behavior of a material is usually determined by comparing the sample thickness to √(a.TTI). When the sample thickness is much higher, the material is considered thermally thick (see for example [11]). Nevertheless, for the reasons discussed above, this approach is not fully satisfying. Indeed, a thick material (borderline case: a semi-infinite material) with a negligible thermal diffusivity behaves as thermally thin (i.e., time-to-ignition is very low and does not depend on heat transfer into the bulk) even if its thermal thickness is much higher than √(a.TTI). In such cases, thermal thickness should be compared not to the whole sample thickness but to the thickness of the limited volume contributing to fuel release leading to ignition.

Another way is to consider the thermal effusivity of the material √a.ρ.C_p_ = √(k.ρ.C_p_) with ρ as the density, k as the thermal conductivity and C_p_ as the specific heat capacity of the material. The thermal effusivity of a material characterizes its ability to exchange thermal energy with its environment. The higher the effusivity, the more energy the material absorbs, without heating up significantly. On the contrary, the lower the effusivity, the faster the material heats up. For a thick layer of foams with low thermal conductivity and low density, the thermally thin behavior accords well with a low effusivity. This definition does not involve the thickness.

Peak of heat release rate (pHRR) is one of the most important parameters to assess the fire hazard and the ratio pHRR/TTI was proposed to measure the flashover propensity of a material [12]. Recently, we have proposed a phenomenological model to calculate the pHRR of thin materials, such as textiles, measured in cone calorimeter from only three parameters:

- the sample mass;
- the external heat flux;
- the effective heat of combustion which is usually close to the heat of complete combustion for most materials [13].

This model is based on the hypothesis that the whole sample mass burns in a brief period. The equation is validated for a set of 36 synthetic and lignocellulosic woven or knitted fabrics but also thin wood or polymer sheets (<2 mm-thick). This model allows the rough prediction of the pHRR but is only suited for thin and thermally thin materials.

Even though most insulating materials are thermally thin (due to their very low effusivity), this model is not suitable for these materials because their thickness is high, and it is not reasonable to consider that the whole sample mass is burnt in a short time. The prediction of the pHRR of such thick but thermally thin materials requires a new model which is the purpose of the present article. Such materials are often biobased while bio-aggregates are more and more used to prepare insulating materials. 

The evaluation of the fire reaction of bio-based insulating materials or other flammable lightweight material was investigated using a cone calorimeter. The influence of the main parameters was identified from a series of tests conducted on bulk flame retardant (FR) and FR-free hemp fibers used as thermal insulating material for which the test protocol was adapted.

From these experiments, a new phenomenological model able to calculate the peak of heat release rate (pHRR) of bulk fibers was proposed. It is based on the fact that the materials investigated are still thermally thin when pHRR occurs (few seconds after ignition, then it is reasonable to consider that heat transfer is still negligible at this time). Thereafter, the model was applied to various classes of thick, but thermally thin, materials as foams, biobased concretes or light woods. All the data were previously gathered mainly from different projects led in IMT Mines Alès.

## 2. Results

The pHRR prediction for insulating materials is based on a simple equation considering only a couple of parameters. These parameters were first identified from the fire behavior of a large set of fibers in bulk and panels (series A and B). Their fire behavior is described in the following section.

### 2.1. Fire Behavior of the Series A and B

**Figure 1:**
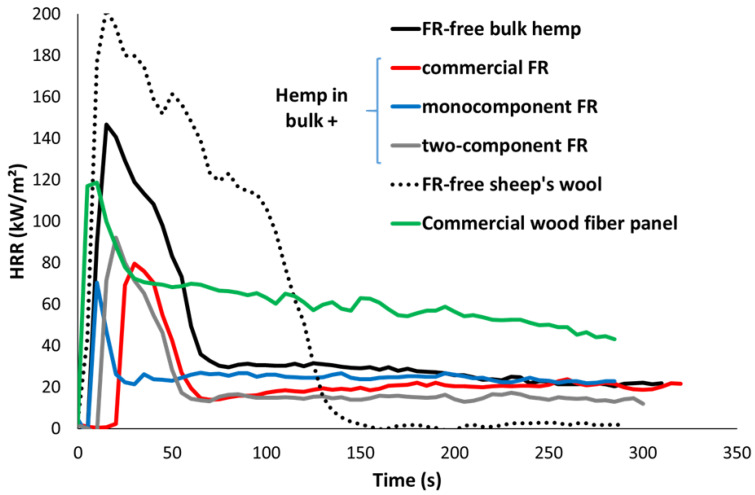
HRR curves in cone calorimeter for various fibers in bulk and panels (heat flux 35 kW/m², no grid).

As already explained, panels and fibers in bulk have a very low density and thermal conductivity. Consequently, their thermal behavior is thin, despite their large dimensions (thickness of several cm). Figure 1 shows some typical HRR curves. Such behavior is characterized by a very low time-to-ignition (TTI), a sharp pHRR occurring few seconds after TTI and followed by a rapid decrease down to extinction. This is in good agreement with the so-called thermally thin behavior described by Schartel and Hull in their classification of the fire behaviors in cone calorimeter [7]. In the case of lignocellulosic materials, the heat release rate does not decrease to 0 kW/m² even after extinction due to thermo-oxidation of char or unpyrolyzed underlying materials. A plateau is observed around 20–30 kW/m² depending on the material. In some cases, the plateau is replaced by a slow decrease at a higher HRR level (see wood panel for instance).

In this study, panels and fibers in bulk exhibit a TTI lower than 25 s (with one exception: sheep’s wool at 25 kW/m^2^ in the presence of a grid, TTI = 48 s), and the pHRR ranges from 30 to 250 kW/m^2^ (slightly higher for sheep’s wool at high heat fluxes without grid). The effective heat of combustion after 250 s ranges from 3.5 to 12 kJ/g for lignocellulosic fibers and panels, depending on the flame-retardant systems. The value is slightly higher (15–16 kJ/g) for sheep’s wool. Note that the values for FR free materials (hemp, wood, sheep’s wool) are close to the heat of complete combustion as reported in the literature [14,15,16].

**Figure 2:**
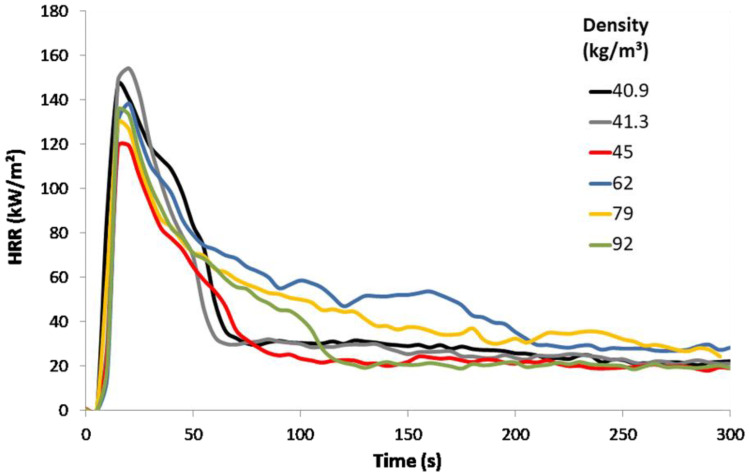
HRR curves in cone calorimeter for FR free bulk hemp fibers for various densities (thickness 70 mm, heat flux 35 kW/m², no grid).

Density of bulk fibers and panels is lower than 100 kg/m³ but differs from one formulation to another. To evaluate the influence of density on the reaction-to-fire, several tests were conducted on FR free hemp fibers in the same conditions but at different densities. Density was modified manually by scattering the hemp fibers in the sample holder (with more or less compaction—the density is believed to be relatively homogeneous, but the homogeneity cannot be accurately assessed). Figure 2 shows the HRR curves for density ranging from 40 to 92 kg/m^3^. It is obvious that the main properties, i.e., TTI and pHRR, do not change regardless of density. The HRR curves present some differences after the pHRR (the level of HRR plateau) but no clear trend is noticeable.

**Figure 3:**
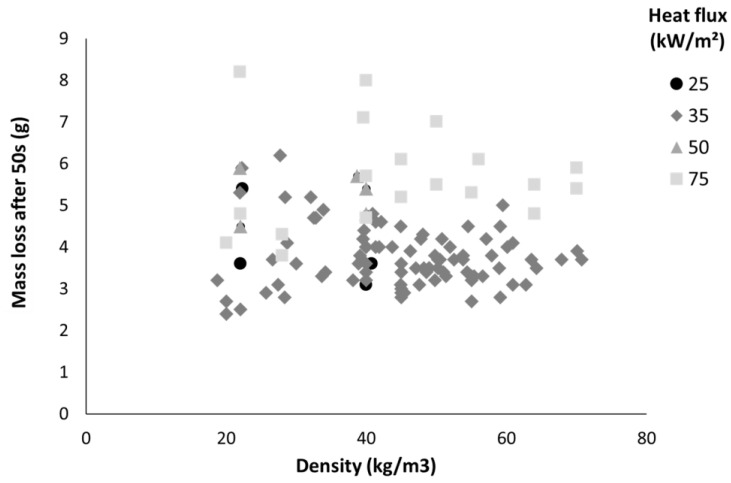
Sample mass loss after 50 s versus their density for series A and B.

The main objective of this work is to predict the pHRR of insulating materials in cone calorimeters. As already explained, pHRR occurs a few seconds after ignition, and it would be helpful to know the main flammability parameters at this stage, i.e., the mass loss and the effective heat of combustion (EHC). Both mass loss and heat of combustion in cone calorimeter can be calculated for each time step. Nevertheless, when the time step is too short, values are not precise and may be inconsistent. A compromise must be found. In this work, the mass loss and the EHC were calculated over a period of 50 s after the beginning of the test. Ignition and pHRR systematically occur during this period. The mass loss after 50 s is plotted versus density in Figure 3. Despite a significant data dispersion, it is clear that the mass loss does not depend on the density and may be considered to be roughly in the range of 3–6 g. The mean value (from all the datapoints) is 4.14 g. As observed in Figure 3, the mass loss at 50 s increases with the heat flux. The mean value is lower than 4 g for 25 and 35 kW/m^2^ and lower than 6 g for 75 kW/m^2^. This is expected because the burning is faster at high heat flux, therefore the mass loss is higher at a given time. The peak of HRR also occurs earlier at high heat flux.

**Figure 4:**
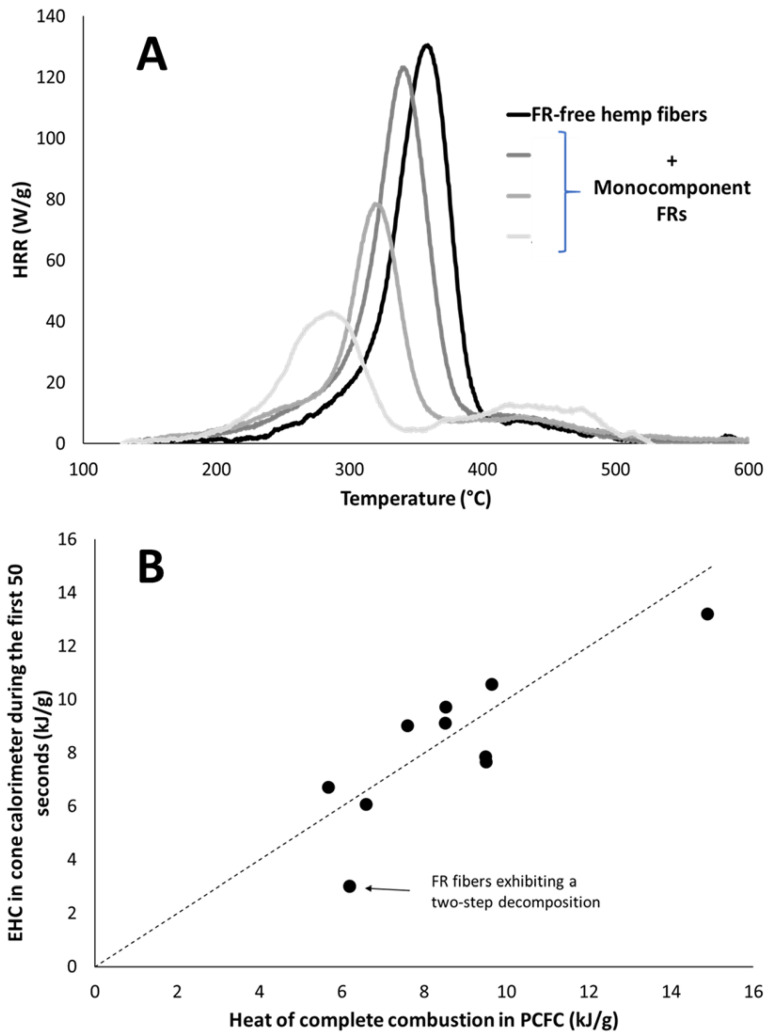
(**A**) HRR curves in PCFC for some raw and flame-retardant hemp fibers; (**B**) Effective heat of combustion (EHC) during the first 50 s in cone calorimeter versus the heat of complete combustion calculated in PCFC (dotted line corresponds to x = y).

The EHC is calculated over the first 50 s. It ranges from 3 to 16.9 kJ/g depending mainly on the additives used to improve the flame retardancy of fibers. This value is generally close to the effective heat of combustion over the whole test. Moreover, while the cone calorimeter is a well-ventilated test, the EHC is often close to the heat of complete combustion measured in pyrolysis combustion flow calorimetry (PCFC), when no flame inhibitor (typically halogen-based compounds) is used. Figure 4A shows the HRR curves from PCFC. FR free hemp fibers decompose in one apparent step centered at 360 °C. Depending on the flame-retardant system, temperature and peak intensity decrease down to around 280 °C and 40 W/g, respectively. A shoulder at higher temperature can be observed and may correspond to the lignin fraction. It is more apparent for flame retarded fibers, surely because the main decomposition step is shifted to lower temperatures.

Figure 4B plots the heat of complete combustion Δh versus the EHC after 50 s for several fibers. It is clear that the EHC is close to the heat of complete combustion in most cases. Nevertheless, there is an exception. Especially when the decomposition occurs in several steps: EHC at the beginning of the cone calorimeter test may correspond to the first step measured in PCFC (see [17] about the change of EHC for binary systems in cone calorimeter). Therefore, a discrepancy can occur between the EHC at 50 s and Δh.

In the original model developed in the study of El Gazi et al. [13], a grid was used to avoid the distortion of some thin fabrics during cone calorimeter tests. Of course, the presence of a grid limits the absorption of heat by the sample and modifies both TTI and pHRR. In order to quantitatively assess the impact of the grid in this present study, several materials were tested in different conditions with and without grid and their pHRR were compared (Table 1).

As shown in Table 1, pHRR without grid is systematically higher than pHRR obtained with a grid and the ratio between both pHRR is relatively constant, around 1.34 (±0.14). Based on these results, the pHRR prediction in presence of a grid (or not) can be estimated by using a constant factor which was fixed to 1.4.

### 2.2. Model Proposed to Predict the pHRR

The model proposed by El Gazi et al. [13] is suitable for thin materials such as fabrics. The pHRR is calculated according to Equation (1):

(1) $$pHRR=EHC\times \alpha \times m^{\beta }$$

where EHC is the effective heat of combustion, m the initial sample mass, and α and β two parameters depending on the heat flux (HF) and established from data obtained by El Gazi et al. (around 120 tests at various HF) according to Equations (2) and (3), respectively.

(2) α=0.0818×HF+3.2427

(3) β=0.0025×HF+0.2197

In the case of thick materials, only a fraction of the sample mass (or volume) is directly impacted by the heat flux. Then, the model becomes inaccurate and overestimates the pHRR. Below the upper first layer interacting with the cone irradiance, underlying material does not absorb radiation heat. Its heating is due to the heat conduction from the surface to the bulk. When samples are insulating materials such as foams, fibers in bulk, panels and so on, the thermal conductivity is very low and then the heat transfer is negligible. However, the pHRR is observed a few seconds after ignition, which occurs shortly after the beginning of the test. The main idea of this work is that only the mass of the sample part absorbing directly the heat flux from the cone (called the absorbing mass m_a_) and not the entirety of the initial sample mass, contributes to the pHRR. Of course, when the material contains a mineral fraction (as for biobased concretes), the corresponding mass contributing to pHRR is the product of the organic fraction f_o_ and the absorbing mass m_a_. 

The pHRR may then be calculated from f_o_, m_a_, the heat flux, the heat of combustion of the organic fraction EHC_o_, and a coefficient specific to the presence of a grid. The extended model to predict the pHRR of thermally thin materials, regardless of their thickness, is given by Equation (4).

(4) $$pHRR={EHC}_{o}\times \alpha \times {\left(f_{o}\times m_{a}\right)}^{\beta }\times C_{Grid}$$

The product $\alpha \times {\left(f_{o}\times m_{a}\right)}^{\beta }$ has the same dimension as a mass loss rate (g/(m^2^.s)). C_Grid_ is a factor depending on the presence of a grid (dimensionless). C_Grid_ = 1 in presence of a grid (2 × 2 cm^2^) and 1.4 without a grid.

The absorbing mass m_a_ is the minimum value between the initial sample mass and the mass loss at 50 s, chosen to be 4.14 g as the mean value determined experimentally for hemp bulk fibers and panels (series A and B). For the lightest materials, for which sample mass is lower than this threshold value, the absorbing mass is equal to the whole initial mass. For thicker materials, a threshold value must be chosen which corresponds to the maximum penetration of radiative flux. In this article, in order to make Equation (4) predictive, this value is considered to be fixed and independent from the material. The mean value for mass loss at 50 s, i.e., 4.14 g from Figure 3, is chosen. This value allows a good fit between the predicted and experimental pHRR values (see below) and seems to be reasonable while pHRR systematically occurs in the first 50 s. A same threshold value for all materials is obviously a rough assumption. It is necessary to check if the absorbing mass varies greatly from one material to another one and changes significantly the predicted pHRR. In Figure 3, the mass loss at 50 s varies between 3 and 6 g. Nevertheless, by considering the coefficients α and β, the pHRR increases only by 10–15% (depending on the heat flux) when the mass increases from 3 to 6 g in Equation (4) (while it increases by 21–33% when the masse increases from 1 to 2 g). Therefore, choosing a fixed value for absorbing mass around 4 g appears to be a good compromise.

EHC_o_ represents the energy which may be released per gram of the organic fraction. For most samples, f_o_ is equal to 1 and EHC_o_ is equal to EHC. As discussed above, EHC after 50 s is generally close to the value measured over the whole test but also to the heat of complete combustion. If the period of time (i.e., 50 s) is arbitrarily chosen as a compromise to calculate the EHC, this choice is considered to be relevant while the experimental pHRR are reasonably predicted (cf. below). Moreover, calculating EHC over a larger period of time does not change the values significantly. For some series, EHC after 50 s is not available, and EHC over the whole test was considered (for series E, F, G, I, J, K, N, P).

In some cases, especially the series H (fabrics) and L (EPS and XPS foams), EHC is not available. Therefore, its value was replaced by 0.9 × ∆h with ∆h the heat of complete combustion. The coefficient 0.9 is used to consider that combustion is not fully complete in cone calorimeter but quite high, because cone calorimeter is a well-ventilated test, and combustion is usually near complete. Note that this approach is valid because combustion efficiency in a cone calorimeter is high. Some flame retarded PU foams from series I contain halogen atoms and their EHC value in cone calorimeter is much lower than their heat of complete combustion. In that case, considering ∆h would lead to a strong overestimation of the pHRR.

When f_o_ is not equal to 1, EHC_o_ may still be calculated from cone calorimeter data (see below for series D). Nevertheless, in other cases, EHC is very noisy and then inaccurate. It is the case for biobased concretes for which the flammability is very low [18]. The pHRR is typically lower than 100 kW/m², flame out occurs in a few dozens of seconds and HRR remains stable at a level close to 20–30 kW/m^2^. In such cases, the heat of combustion of the organic fraction is also considered to be equal to 0.9 ×∆h_o_ (∆h_o_ being the heat of complete combustion of bioresource measured in PCFC).

It is important to understand that in all these cases, the objective is to estimate conveniently the heat which can be released by the material. Table 2 indicates how this heat was measured for every series.

### 2.3. Assessment of the Model

The main hypothesis of the model is that the pHRR is dependent on the mass directly absorbing the heat flux from the cone calorimeter, while the underlying layer does not contribute to the pHRR due to negligible heat transfer. As mentioned previously, the absorbing mass was roughly constant, and its value was close to 4.14 g as experimentally measured from hemp bulk fibers and panels (series A and B). The numerical model introduced in the materials and methods section was implemented to check if such hypothesis is relevant.

**Figure 5:**
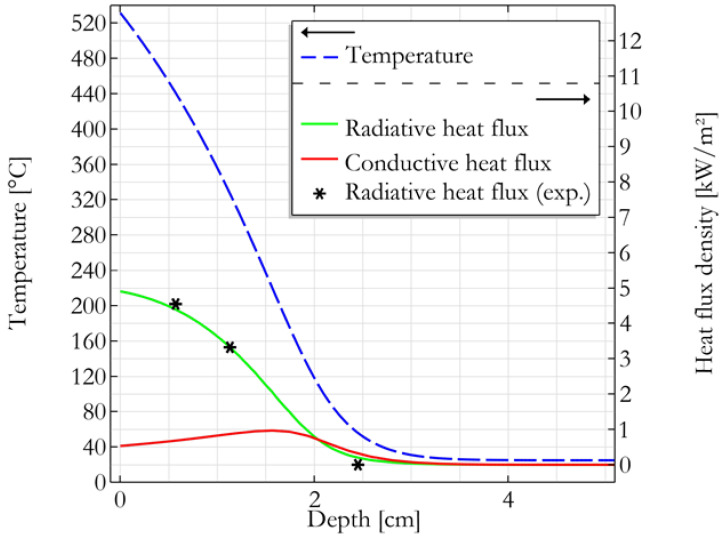
Simulation of the temperature and heat fluxes distributions in a panel as a function of depth along the cone axis after 50 s in a cone calorimeter at 35 kW/m².

The radiative component of the heat flux (cf. modified Fourier’s law in Equation (8)) resulting from the simulation was plotted for different extinction coefficient values. The value of 450 m⁻¹ leading to the best fit with the experimental data is considered thereafter for the extinction coefficient, as shown in Figure 5. Note that Linteris et al. measured absorption coefficients for several dense polymers and found values from 1000 to 6000 m⁻^1^ [19]. But these data correspond to bulk polymers while our materials are highly porous. However, the extinction coefficient is the sum of the absorption and scattering coefficients. Therefore, scattering may explain why the extinction coefficient for the hemp panel is only 2 to 13 times smaller than the values reported by Linteris et al., while the density is at least 20 times lower.

**Figure 6:**
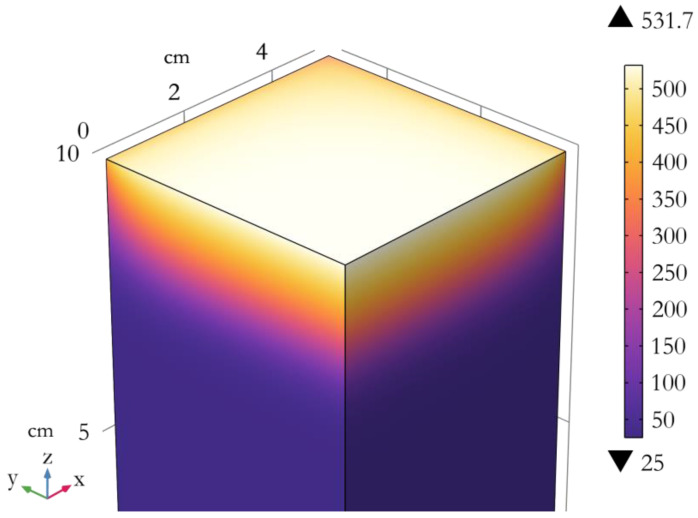
Simulation of the temperature distribution in a quarter of a panel (visible vertical faces correspond to symmetry planes) after 50 s in cone calorimeter at 35 kW/m².

Figure 5 also confirms the hypothesis that conductive heat flux density is much lower than its radiative counterpart, especially in the first centimeters of the material. Figure 5 exhibits the temperature distribution along the axis of the cone. Heat seems to be kept mostly at the top of the panel and a high temperature gradient is visible in the first 2 cm. Figure 6 shows the temperature distribution in the rest of the sample. For a given height, the temperature turns out to be rather homogeneous except at the boundaries where heat is released by radiation.

As a reminder, the mass loss is supposed to be around 4.14 g. According to the simulation, the volume corresponding to 4.14 g is at a temperature higher than 320 °C. This temperature would be the minimum temperature that fibers should reach to contribute to pHRR. This temperature threshold can be compared to the thermal stability of fibers as measured in PCFC (see Figure 4A). For FR free hemp fibers, the decomposition occurs roughly in the range of 250–400 °C. For flame-retarded fibers, the decomposition starts earlier (close to 200 °C), but the peak temperature is close to 280–290 °C. Therefore, a temperature of 320 °C seems a reasonable estimation of the temperature of fibers contributing to the decomposition at an early stage, i.e., for pHRR.

**Figure 7:**
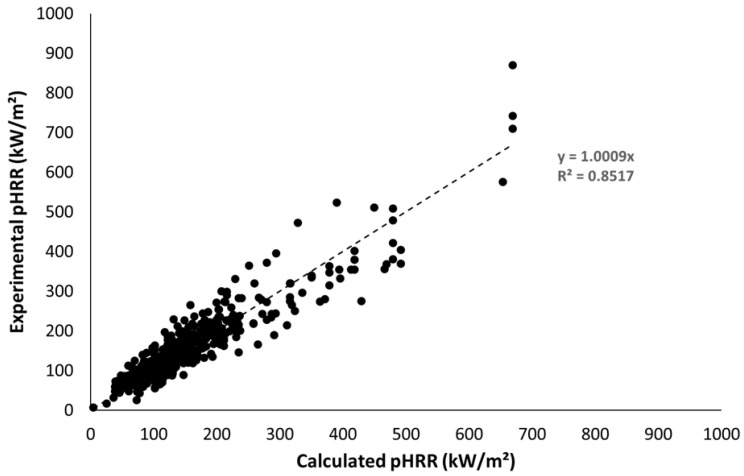
Experimental versus calculated pHRR for all materials tested.

Equation (4) was tested for all series, i.e., more than 400 tests in various conditions and for different classes of materials. All the data are listed in Supplementary Materials. Figure 7 plots the experimental pHRR versus the calculated pHRR from Equation (4). The slope of the fitting curve is almost 1 and the coefficient of correlation is quite satisfying (R^2^ = 0.85). The mean experimental pHRR over all the series is 163 kW/m^2^ and the absolute mean error on prediction (calculated according to Equation (5)) is 18.1%.

(5) $$Absolute\;mean\;error=\frac{1}{n}\times \sum_{n}100\times \frac{\left|{pHRR}_{exp}-{pHRR}_{calc}\right|}{{pHRR}_{exp}}$$

where ${pHRR}_{exp}$ is the experimental pHRR and ${pHRR}_{calc}$ the calculated pHRR.

**Figure 8:**
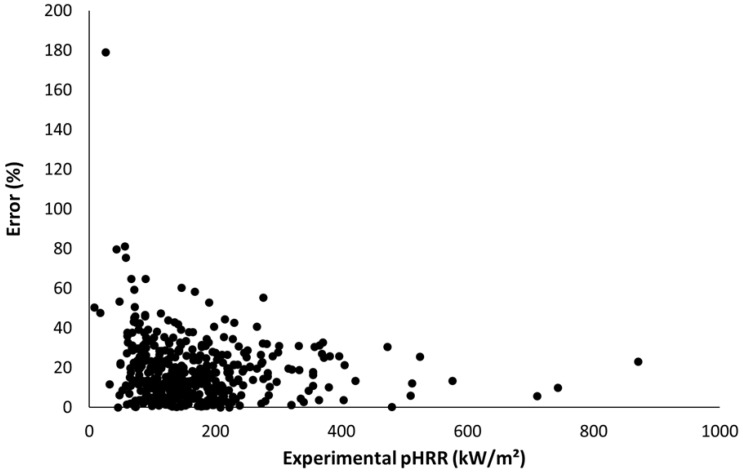
Absolute error versus experimental pHRR.

Figure 8 shows the error versus the experimental pHRR for all datapoints. In total, 50% of tests exhibit a error lower than 16%. The mean error is increased for materials exhibiting low pHRR (13 tests lead to an error higher than 50%, including 9 tests for which the experimental pHRR is lower than 80 kW/m^2^). Therefore, the mean error can be considered satisfying. In ISO 5660 standard [20], results from a set of interlaboratory trials are reported and allow us to calculate the repeatability. For a material exhibiting a peak of 300 kW/m^2^, the repeatability is estimated to be 53 kW/m^2^ (i.e., 17.7% of the pHRR). The repeatability is 30% when the pHRR is as low as 80 kW/m². Similarly, the reproducibility is 103 and 70 kW/m^2^ (i.e., 34 and 89% of the pHRR) for materials exhibiting, respectively, pHRR of 300 and 80 kW/m^2^.

**Figure 9:**
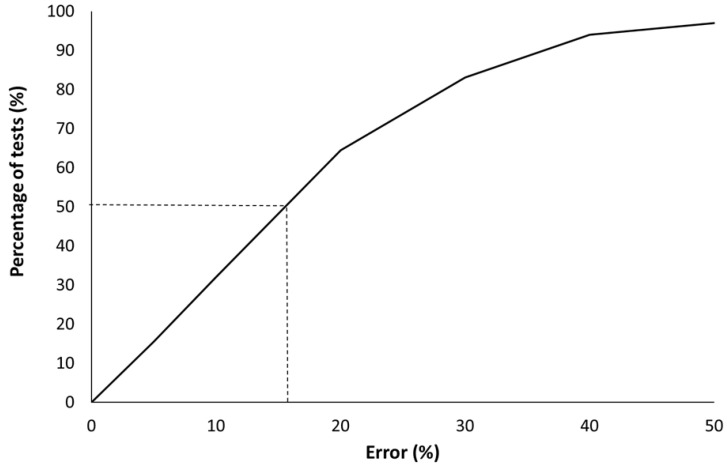
Percentage of tests for which the error is smaller than the indicated value (for example, 50% of the tests exhibit an error lower than 16%).

Figure 9 shows that 32.1% of the 430 tests have an error lower than 10%, 64.2% have an error lower than 20%, and 82.8% have an error lower than 30%. Therefore, it can be assumed that the model allows a rough estimation of the pHRR of insulating materials from only a couple of easily available parameters.

**Figure 10:**
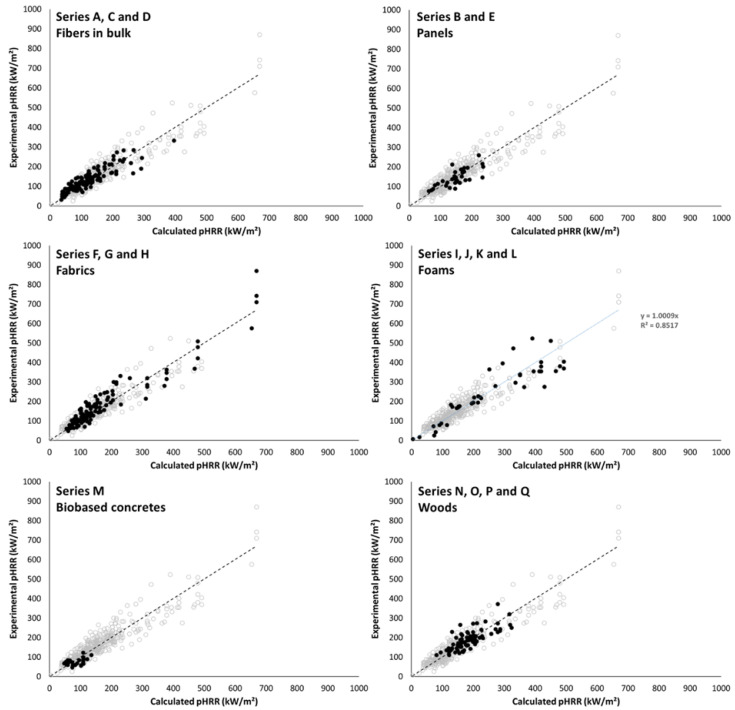
Experimental versus calculated pHRR for all series (for each figure, black points correspond to the considered series).

Figure 10 highlights the datapoints corresponding to the different series of materials, i.e., fibers in bulk, panels, fabrics, foams, biobased concretes and woods.

Series A, C and D include fibers in bulk (mostly lignocellulosic fibers). The mean experimental pHRR is 134 kW/m^2^, and the absolute mean error is 18.6%. Despite a great variation of conditions (sample mass between 8 and 45 g, thickness between 25 and 70 mm, heat flux between 25 and 75 kW/m^2^, presence of a grid or not), the prediction accuracy is correct. The series D corresponds to needles from pinus pinea species flame-retarded by a known amount of aqueous solution of ammonium phosphate. The solution was sprayed on needles before the test but the dispersion of the FR on needles is not controlled. However, the ability of FR to modify the charring and the heat of combustion of needles depends on its dispersion on needles. Therefore, measuring ∆h_o_ of needles with a specific amount of FR does not warrant that this value is close to EHC_o_. For this series, EHC_o_ used in Equation (4) was estimated from the fraction of water w, the fraction of needles f_o_ (considering f_o_ + w = 1) and the effective heat of combustion EHC measured in a cone calorimeter using Equation (6).

(6) $${EHC}_{o}=EHC\times \left(\frac{f_{o}+w}{f_{o}}\right)$$

For series D, the mean pHRR is 198 kW/m^2^ over 13 tests and the absolute mean error is 17.9%, evidencing that such calculation of EHC does not deteriorate the prediction.

For panels (series B and E), tests were mainly carried out at 35 kW/m^2^ (but some additional samples were tested at 25, 50 or 75 kW/m^2^). Experimental pHRR is 145 kW/m² and the absolute mean error is 19.4%, i.e., very similar to the values for fibers in bulk.

**Figure 11:**
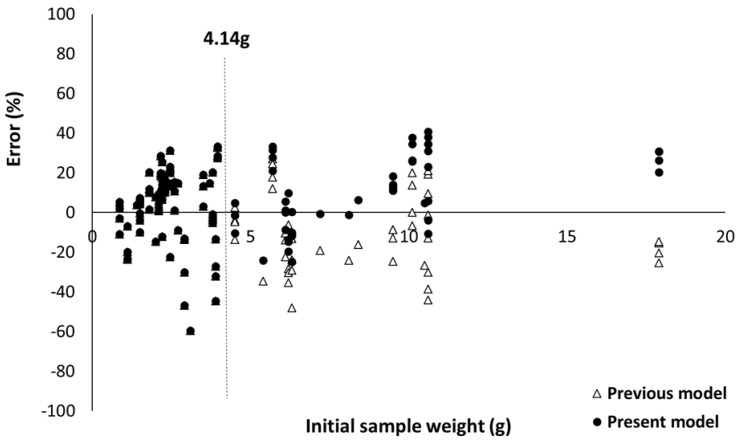
Absolute error for fabrics versus sample weight according to the present model and the previous one (El Gazi [13]).

Series F, G and H include synthetic or natural fabrics tested at a heat flux ranging from 25 to 75 kW/m^2^. Some of these fabrics were mixes (wool/nylon, wool/cotton, wool/flax and so on). Most of the fabrics are very thin samples with an initial mass lower than the absorbing mass m_a_ (i.e., 4.14 g). The experimental pHRR is 181 kW/m^2^ and the absolute mean error is 15.7%. The previous model proposed by El Gazi et al. [13] was developed for such materials. The comparison of the predictions obtained from this model and the present one for series F, G and H is shown in Figure 11. The relative error for each test was calculated with Equation (7).

(7) $$Relative\;error=100\times \frac{{pHRR}_{exp}-{pHRR}_{calc}}{{pHRR}_{exp}}$$

where ${pHRR}_{exp}$ and ${pHRR}_{calc}$ are experimental and calculated (predicted) pHRR, respectively.

Of course, for an initial mass lower than 4.14 g, both models lead to the same predictions. For higher initial mass, the previous model overestimates the pHRR (${pHRR}_{exp}$<${pHRR}_{calc}$), while the present model tends to underestimate it. The absolute mean error (Equation (5)) is slightly lower for the present model (15.7% versus 16.3%). Note that the predictions are not only correct for the samples tested by El Gazi et al. [13] (i.e., the fabrics from which the model was developed) but also for the fabrics studied by Morgan et al. [21,22].

Series I, J, K and L (foams) gather highly flammable foams (FR-free flexible PU or PS foams) and highly flame-retarded foams (FR rigid PU foams and alginate foams, exhibiting an excellent fire behavior even without flame retardant). Collapse may occur during burning for some foams, changing the heat release rate. Nevertheless, the shape of HRR curves is compatible with the so-called thermally thin behavior and therefore these data were used in the present work. Tests were carried out at various heat fluxes (from 17 to 75 kW/m^2^). The experimental mean pHRR is 229 kW/m^2^, and the absolute mean error is higher (25.7%). This is especially due to some low flammability alginate-based foams for which the error is in the range of 47–179%. As an example, one of these foam burns for a few seconds with a pHRR of 8 kW/m^2^. The predicted pHRR is 4 kW/m^2^, i.e., very close, but the error is 50%. Excluding these four tests leads to a more satisfying error (17.2%).

Series M gathers light biobased concretes based on various binders (earth, gypsum and a mix of both) and bioresources (hemp, straw and rice husk). Most tests were carried out at 50 kW/m^2^ but some additional tests were performed at heat fluxes ranging from 30 to 70 kW/m^2^. The bioresource fraction ranges from 0.18 to 0.36. For such low density, thermal conductivity is typically lower than 0.1 W/(m.K). All the considered concretes ignite briefly with a low pHRR (below 125 kW/m^2^, most often below 100 kW/m^2^). The mean pHRR is 77 kW/m^2^. Calculated pHRR are well in the same range (40–140 kW/m^2^). The absolute mean error is high: 28.8%. This apparent high error may be explained by several reasons:

- First, due to the low pHRR, a moderate absolute error results in a significant relative error (for example, an absolute error of 15 kW/m^2^ corresponds to a relative error of 21% when the pHRR is 70 kW/m^2^);
- second, these samples may be less homogeneous than other samples, therefore, the organic fraction at the surface may be slightly different from the fraction calculated over the whole volume;
- third, these materials contain several phases with different thermal stability and endothermic heat related to their pyrolysis or dehydration: the whole decomposition process may be more complex;
- finally, some additional effects are not considered by the model, especially that some plant fibers straighten under heating which increases the exposure of their surface to heat.

Series N, O, P and Q gather various woods from balsa (density lower than 200 kg/m^3^) to some tropical dense woods (density higher than 1000 kg/m^3^). Note that two peaks are observed on HRR curves for thick wood samples (see for example [14]). The second one is an artefact occurring when the heat front reaches the sample bottom at the end of the test [7]. It may be higher than the first one. Of course, our model is proposed to predict the first one occurring just after ignition. The mean pHRR is 191 kW/m^2^, and the absolute mean error is 13.6%. The prediction is good even for denser woods exhibiting relatively high thermal conductivity. This is apparently in contradiction with the hypothesis on which the model is built, i.e., that heat transfer by conduction is negligible. Nevertheless, it should be kept in mind that wood is able to char. The char layer allows the limitation of the radiative heat transfer from the flame to the underlying material. Therefore, the pyrolysis zone becomes thinner after the formation of the char layer even though heat can be transferred by conduction from the surface to the bulk. However, the thickness of this zone controls the mass loss rate and ultimately the HRR. Thus, HRR reaches its peak at the beginning and is quickly reduced to a pseudo-plateau (HRR decreases continuously as long as the heat front progresses down to the sample’s bottom). For such materials, if the radiative heat flux is reduced by a char barrier layer before the heat conduction is effective, the HRR is effectively reduced, and the pHRR is observed in a few dozens of seconds after ignition.

## 3. Discussion

The phenomenological model proposed here seems to allow a rough but satisfying prediction of pHRR for a broad range of different insulating materials, including fabrics, foams, biobased concretes, fibers in bulk, panels and woods. Despite their differences, all these classes of materials (except dense woods) gather similar features. Indeed, they are thermally thin, with a short ignition and a sharp pHRR followed by a rapid HRR decrease.

The model is an extended (but compatible) version of the previous model presented by El Gazi et al. [13] adapted for textiles and other thin materials. It is based on the fact that only the top layer absorbs the external radiative heat flux and contributes to the pHRR. This top layer screens the underlying material which is heated neither by the direct radiation nor by heat transfer due to the low thermal conductivity. The screening mass is roughly constant (around 4 g). This is obviously an approximation but our results on a large set of materials evidence that this approximation is relevant for a fast and rough calculation of pHRR.

**Figure 12:**
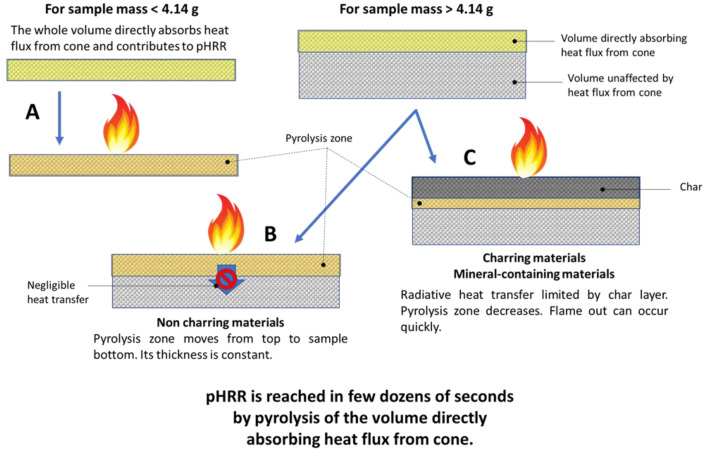
Scheme describing the fire behavior of the materials tested in this study (A, B and C correspond to the three cases for which Equation (4) is suitable).

Figure 12 summarizes the different scenarii observed in this study:

- Scheme A: for very thin samples with an initial mass lower than approximately 4 g, such as most fabrics, the whole sample absorbs the heat flux from the cone calorimeter and is heated. There is almost no temperature gradient through the thickness. The whole mass contributes to the pHRR according to Equation (4). Note that, in that case, some flame-retardant strategies as the so-called barrier effect should not be effective, because the protective barrier is formed too late.
- Scheme B: for heavier but insulating samples, only a small part of sample (around 4 g) directly absorbs the heat flux from the cone calorimeter. The thermal conductivity is negligible, and then the underlying material is not heated by heat transfer (or this heating is too slow). Then, only the mass directly absorbing the heat flux contributes to pHRR. The HRR is reduced after pHRR because the residue layer (char in the case of lignocellulosic fibers or FR foams, or mineral fraction in the case of biobased concretes) blocks the radiative heat flux. When no residue is left, HRR may also be reduced because the sample surface regresses and the distance between the sample and the radiative cone increases. But the HRR reduction should be more limited. Indeed, in the case of non-charring PS foams, a stabilization rather than a sharp reduction in HRR is observed after pHRR (series L [23]).
- Scheme C: the pHRR of dense woods can also be predicted despite a significantly higher thermal conductivity. The reason may be that the reduction in radiative heat flux by the char formation dominates the heat conduction, resulting in a sharp decrease in HRR after few dozens of seconds. Note that such mechanism may also be expected for other classes of materials as charring polymers or polymers filled with high amounts of mineral fillers. Further investigations are needed to confirm this point. Even though these materials are not strictly thermally thin, the formation of this char, if it is fast enough, leads to a pHRR controlled mainly by the heat flux penetration depth. Therefore, our model can be still successfully used.

The model is based on a very limited set of parameters:

- two related to the test conditions (heat flux and presence/absence of grid),
- one related to the sample dimensions (initial mass),
- one related to the material (heat of combustion).

Of course, such a simple model cannot consider all phenomena occurring during the test that are prone to modify more or less the material response to the heat flux. Therefore, the accuracy of the prediction may be reduced by several limitations listed below.

EHC should ideally correspond to the period over which pHRR occurs, i.e., at the beginning of the test. Unfortunately, for practical reasons, EHC cannot be properly measured over a very short period of time. EHC can change over the whole test. Indeed, some phenomena may change significantly the EHC during the test. Especially, for hydrophilic materials like lignocellulosic ones, the presence of water released at low temperature may impact the change in EHC during the test. Same results are expected in the case of mineral binders containing hydrated minerals (as gypsum or lime in biobased concrete). For most of the materials tested in this study, it may be reasonably assumed that the EHC is close to the heat of complete combustion. Nevertheless, in the case of incomplete combustion, this should not be true. For some FR rigid PU foams, the EHC (used for the calculation of pHRR) is far lower than the heat of complete combustion (around 6 versus 24 kJ/g) due to the presence of chlorine-containing flame retardant [24].

Other phenomena which are not considered by the model can occur, such as the straightening of fibers in some biobased concretes or the collapse of thick flexible PU foams. This is why thick PU foams from the work of [25] were excluded from this study.

Finally, it should be noted that this model is unable to predict the ignition of samples. For example, some biobased concretes close to those considered in series M are not ignited despite the fact that their pHRR predicted by Equation (4) can reach similar values. Contribution of the mineral matrix in the fire behavior of such composite must be clarified (release of water at 150 °C from gypsum or 450 °C from hydrated lime for example).

## 4. Materials and Methods

The model predicting the pHRR is developed from a series of fibers (in bulk) and fiber panels characterized in cone calorimeter at various heat fluxes. These series (respectively, called series A and B—see Table 2) include:

- Overall, 26 bulk hemp fibers treated with commercial or lab-made flame-retardant systems—their density in sample holder is in the range of 40–60 kg/m³ depending on the formulation;
- a total of 6 additional bulk fibers, including one from animal source (sheep’s wool)—their density is lower, in the range 20–30 kg/m³;
- and 5 panels from various lignocellulosic fibers—their density ranges from 20 to 55 kg/m^3^.

**Table 2:** List of samples considered in the present study.

Series	Samples	Number of Tests	$\mathrm{Calculation}\;\mathrm{of}\;{EHC}_{o}$	Reference
A	Lignocellulosic or sheep FR and FR-free fibers in bulk	100	Over the first 50 s	/
B	Panels mainly from lignocellulosic FR and FR-free fibers	15	Over the first 50 s	/
C	Several biobased fibers in bulk mixed with a mineral component	6	$0.9\times {∆h}_{f}$ *	/
D	Needles in bulk flame retarded with an aqueous solution avec ammonium phosphate	13	${EHC}_{o}\times f_{o}$ *	/
E	Additional panels from hemp or Posidonia	17	Over the whole test	/
F	Fabrics	108	Over the whole test	[13]
G	Fabrics	12	Over the whole test	[21]
H	Fabrics	5	0.9×∆h	[22]
I	Polyurethane (PU) or alginate foams (some of them have been published)	15	Over the whole test	[24,25]
J	PU foams	6	Over the whole test	[26]
K	PU foams	12	Calculated from cone data	[27]
L	Expanded and extruded and Polystyrene (EPS and XPS) foams	8	0.9×∆h	[23]
M	Biobased concretes (some of them have been published)	31	$0.9\times {∆h}_{f}$	[18]
N	Woods of different natures and densities	30	Over the whole test	/
O	Woods of different natures and densities	37	0.9×∆h	/
P	Mexican woods (tested surface area 7 × 7 cm²)	7	Over the whole test	[28]
Q	Woods	7	0.9×∆h	[14]

* ${EHC}_{o}$ is the effective heat of combustion of the fuel fraction, ${∆h}_{f}$ is the heat of complete combustion of fibers, $f_{o}$ is the fuel fraction, ∆h is the heat of complete combustion of the whole material (the use of these parameters is discussed in the text below).

**Figure 13:**
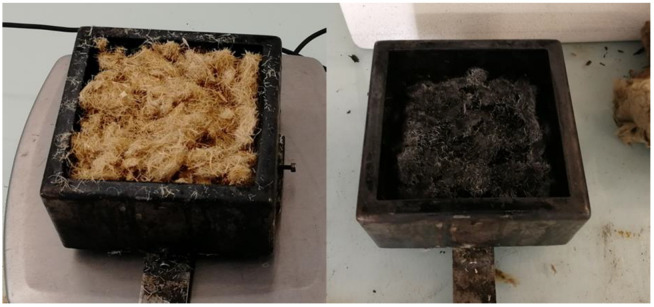
Untreated hemp fibers scattered in the sample holder before (**left**) and after (**right**) cone calorimeter test.

Usually, these materials were assessed using a cone calorimeter at 35 kW/m^2^ according to ISO 5660-1 [20]. This heat flux is very common and corresponds to a developing fire [7]. For bulk fibers, the sample holder was filled by cautiously scattering fibers as to reach a given thickness (up to 70 mm, cf. Figure 13). Even if the distribution of fibers is not perfectly homogeneous, the density is considered to be rather constant through the whole thickness (the same density was obtained when the sample holder was filled at different depths with the same fibers). Panel thickness was fixed to 55 mm. Additional tests were performed to check the suitability of the model in various conditions: heat flux ranged from 25 to 75 kW/m^2^, the sample thickness was reduced in order to test a thinner bed of fibers, and a grid (2 × 2 cm^2^, rod diameter 1 mm) was placed above the sample in some cases (grid is generally used to avoid sample distortion, especially for textiles—Note also that, in the case of bulk fibers, the grid is cautiously placed on the specimen in order to avoid any compaction of fibers). Fibers were tested after storage in the cone calorimeter room during several weeks (temperature and relative humidity (RH) were maintained around typically 20–25 °C and 15–30%, respectively).

The flammability of several fibers was also characterized at microscale using the pyrolysis-combustion flow calorimetry according to ASTM D-7309 method A (anaerobic pyrolysis), especially to measure the heat of complete combustion (as the ratio between the total heat release and the fraction of mass loss). Small samples (few milligrams) were burnt in a pyrolizer going up to 750 °C at 1 °C/s under nitrogen flow. Gases released from pyrolysis were sent to a combustor heated at 900 °C under air flow (N_2_/O_2_ = 80/20). Combustion is considered to be complete in these conditions. Heat release rate (HRR) was determined according to oxygen depletion (Huggett’s relation [29] as in cone calorimeter test).

In the case of thermally thin but thick materials, the proposed model assumes that the burning of a limited sample mass is involved at pHRR. To check the reliability of this assumption, a three-dimensional numerical model was implemented with COMSOL Multiphysics^®^ software in order to evaluate the temperature distribution in one sample (namely, the flame retardant-free hemp panel with a density of 43 kg/m^3^, series B) during a cone calorimeter test. By considering the planar symmetries of the problem and the boundary conditions, the model was simplified as a quarter of the real geometry (i.e., a block of 5 × 5 × 10 cm^3^). The initial and ambient temperatures were considered to be 25 °C. A cone irradiance of 35 kW/m² was applied to the top surface, considering its heterogenous distribution according to Wilson et al. [30] and a distance of 25 mm between the surface and the base of the cone. The top surface was also considered to undergo a convective heat flux with ambient air assuming a heat transfer coefficient of 10 W/(m^2^/K). Top and lateral faces were considered to release radiative heat with the environment according to the Stefan–Boltzmann law. Symmetry planes were considered fully insulated. In order to consider the penetration of the cone irradiance in the first centimeters of the sample, the latter was considered as an optically thick semi-transparent media. In order to achieve reasonable computation times, the Rosseland approximation was implemented. It considers that light propagation behaves similarly to heat transfer (when the optical depth is significantly higher than 1) and adds a “radiative” term into the Fourier’s law according to Equation (8).

(8) $$q=-k\nabla T-\frac{16\sigma n^{2}T^{3}}{3\beta_{R}}\nabla T$$

where q is the total heat flux density, T the temperature, k the thermal conductivity, n the refractive index, β_R_ the extinction coefficient and σ the Stefan–Boltzmann constant.

The mesh was made of hexahedral elements with quadratic Lagrange interpolation. Their dimension was 5 × 5 mm^2^ in the horizontal directions and a linear growth rate was applied in the vertical direction from top to bottom for a total of 40 elements. The time-dependent study was solved up to 50 s with steps of 1 s.

As said above, thermophysical properties of the hemp panel are needed for the numerical model. Emissivity was fixed to 0.9 and assumed to be independent from the temperature.

The thermal conductivity of the hemp panel was assessed with the transient line-source probe method. A FP2C apparatus from Neotim (Albi, France) was used. Materials were stored at 23 °C and 50 %RH during 4 h. A heating source of 0.05 W was applied during 90 s and the subsequent temperature increase was recorded to evaluate the thermal conductivity. The final value of 38 mW/(m.K) results from the average of 3 measurements. This value was used in the simulation assuming temperature independence.

The specific heat capacity of hemp panels was assessed with a calorimeter. A C80 (Calvet) apparatus from Setaram (Caluire-et-Cuire, France) was used. A temperature ramp of 0.2 K/min from 10 to 100 °C was applied to two cells in one of which the 2.85 g sample was placed. The sample was previously dried for 8 h at 80 °C under vacuum. The measurement of the difference of the heat fluxes entering each cell (sample and reference) allowed the determination of the specific heat capacity of the sample as a function of temperature. Between 20 and 96 °C, the specific heat capacity increases mostly linearly from 1.21 to 1.61 J/(g.K). The thermo-dependence of the heat capacity was implemented in the simulation considering linear interpolations between data points and a constant extrapolation above 96 °C.

In order to assess the extinction coefficient value, the fluxmeter was positioned under the radiant cone to measure a heat flux of 12 kW/m^2^. Hemp panel samples of several thicknesses (i.e., 0.57, 1.13, and 2.45 cm) were placed between the radiant cone and fluxmeter, and the heat flux was measured after a few seconds in order to assess the attenuation of the heat flux through the sample.

Finally, pHRRs were calculated for several other series of materials, including fabrics, biobased concretes, foams and woods. They were compared to the experimental values in order to check the suitability of the model. Most of the samples were tested at a PCH lab for various projects. Nevertheless, when available, detailed data from published articles were also used to check relevance of the model. The different series (from series C to series Q) are listed in Table 2. The total number of tests considered in this study is higher than 400 (430 exactly). All these samples, with the exception of some woods (discussed below), have a very low thermal conductivity (typically lower than 0.1 W/(m.K)) and low density (i.e., low thermal effusivity), in agreement with our main hypothesis: heat transfer may be neglected in the first dozens of seconds when pHRR occurs.

## 5. Conclusions

The thermally thin behavior of lightweight materials exhibited during cone calorimeter tests (at least during a period including the occurrence of the pHRR) is a consequence of their low effusivity. Only a thin top layer exposed to the heat flux contributes to the pHRR. The deeper part of the specimen remains at an insufficient temperature to contribute to heat release. The phenomenological model previously developed by El Gazi et al. to calculate the peak of heat release rate of thin materials had to be adapted.

The thickness of the top layer of the sample contributing to pHRR was estimated from experimental data conducted on bulk FR and FR-free hemp fibers. This top layer is believed to directly absorb the heat flux from the radiant cone. Its thickness was evaluated with a numerical model. The corresponding sample mass loss recorded during this period (i.e., the first 50 s) does not appear to be influenced by the density. A mean mass loss of around 4.1 g was evaluated.

Assuming that the total burning of this mean mass leads to the peak of heat release rate, an adjustment of the model is proposed. The incidence of the presence of a grid above the sample is also considered.

Thereafter, the results of the proposed model were compared with experimental results obtained on various types of thermally thin materials in order to assess the relevance of the model. The accuracy of the model appears satisfactory. The model is well appropriate in the case of bio-resources like bulk fibers used as insulating materials for construction. The model was also applied to some limit cases like dense woods (for which the more pronounced heat transfer due to a non-negligible thermal conductivity is prevented by a char development) and biobased concretes (for which the mineral fraction must be considered in Equation (4) through the parameter f_o_).

If the model is based on some rough assumptions, it allows us to readily calculate the pHRR of a large set of materials with an acceptable accuracy from easily accessible parameters.

## Figures and Tables

**Table 1:** Comparison of pHRR with and without a grid for a couple of materials.

Sample	Thickness (mm)	Initial Mass (g)	Heat Flux (kW/m²)	pHRR (kW/m²)		Ratio
				without Grid	with Grid	
Hemp fiber	70	≈25	35	150	125	1.20
	70		50	181	142	1.27
	70		75	232	178	1.30
Sheep’s wool fiber	70	≈14	25	186	111	1.68
	70		35	208	165	1.26
	70		50	284	211	1.35
	70		75	333	238	1.40
Hemp fiber treated with commercial FR	27	≈10	35	143	104	1.38
	70	≈28	35	149	115	1.30
	70		75	219	189	1.16
Hemp fiber treated with lab-made FR	70	≈36	75	176	122	1.44
				Mean value		1.34
				Standard deviation		0.14

## Data Availability

All data used to draw Figure 7 are listed in Supplementary Materials. Raw data are available on request.

## Supplementary Materials

The following supporting information can be downloaded at: https://www.mdpi.com/article/10.3390/molecules28135175/s1, Cone calorimeter data for all series studied.
