# Supplementary material for: Flammability of Thick but Thermally Thin Materials including Bio-Based Materials
Source: Molecules. 2023 Jul 3;28(13):5175. doi: 10.3390/molecules28135175 (PMC10343274; doi:10.3390/molecules28135175)
Supplement: Supplementary file 1 [file molecules-28-05175-s001.zip › molecules-2470760-supplementary.pdf]

Sample	Mass (g)	Heat flux (kW/m²)	Grid (1=yes)	Heat of combustion (kJ/g)	fo	Absorbing mass (g)	Alpha	Beta	Predicted pHRR (kW/m²)	Exp. pHRR (kW/m²)	Error (kW/m²)	Error (%)
Series A												
A-1	25.5	35	0	9.1	1	4.14	6.1057	0.3072	120	147	27	18.1
A-2	25.8	35	0	8.6	1	4.14	6.1057	0.3072	114	154	40	26.1
A-3	25	35	0	9.6	1	4.14	6.1057	0.3072	127	148	21	14.2
A-4	37.5	35	0	3	1	4.14	6.1057	0.3072	40	70	30	43.3
A-5	36.9	35	0	2.98	1	4.14	6.1057	0.3072	39	73	34	46.0
A-6	40.1	35	0	4.1	1	4.14	6.1057	0.3072	54	70	16	22.5
A-7	39.2	35	0	2.9	1	4.14	6.1057	0.3072	38	60	22	36.1
A-8	31.1	35	0	5.9	1	4.14	6.1057	0.3072	78	81	3	3.7
A-9	32.8	35	0	7.5	1	4.14	6.1057	0.3072	99	81	18	22.5
A-10	30.1	35	0	10.2	1	4.14	6.1057	0.3072	135	131	4	3.0
A-11	27.3	35	0	8.9	1	4.14	6.1057	0.3072	118	128	10	8.0
A-12	31.3	35	0	9.1	1	4.14	6.1057	0.3072	120	126	6	4.5
A-13	28.3	35	0	11.4	1	4.14	6.1057	0.3072	151	133	18	13.4
A-14	30.6	35	0	8.1	1	4.14	6.1057	0.3072	107	123	16	12.9
A-15	29.4	35	0	8.7	1	4.14	6.1057	0.3072	115	118	3	2.5
A-16	34.6	35	0	7.1	1	4.14	6.1057	0.3072	94	91	3	3.2
A-17	31.1	35	0	6.4	1	4.14	6.1057	0.3072	85	89	4	4.9
A-18	30	35	0	8.7	1	4.14	6.1057	0.3072	115	146	31	21.2
A-19	24.8	35	0	9.3	1	4.14	6.1057	0.3072	123	142	19	13.4
A-20	30.3	35	0	8.4	1	4.14	6.1057	0.3072	111	110	1	1.0
A-21	29.7	35	0	8.6	1	4.14	6.1057	0.3072	114	110	4	3.4
A-22	28.9	35	0	10.7	1	4.14	6.1057	0.3072	142	153	11	7.5
A-23	28	35	0	9.4	1	4.14	6.1057	0.3072	124	151	27	17.7
A-24	42.4	35	0	4.5	1	4.14	6.1057	0.3072	60	80	20	25.6
A-25	36.9	35	0	6.5	1	4.14	6.1057	0.3072	86	77	9	11.6
A-26	23.8	35	0	6.2	1	4.14	6.1057	0.3072	82	79	3	3.8
A-27	24.4	35	0	6.3	1	4.14	6.1057	0.3072	83	85	2	2.0
A-28	24.3	35	0	9.7	1	4.14	6.1057	0.3072	128	125	3	2.6
A-29	24.4	35	0	9.1	1	4.14	6.1057	0.3072	120	137	17	12.2

A-30	33.6	35	0	2.9	1	4.14	6.1057	0.3072	38	49	11	21.7
A-31	38	35	0	3.4	1	4.14	6.1057	0.3072	45	45	0	0.1
A-32	31.5	35	0	2.7	1	4.14	6.1057	0.3072	36	32	4	11.6
A-33	35.3	35	0	3.4	1	4.14	6.1057	0.3072	45	48	3	6.3
A-34	30.5	35	0	3.9	1	4.14	6.1057	0.3072	52	58	6	11.1
A-35	29.8	35	0	3.2	1	4.14	6.1057	0.3072	42	68	26	37.8
A-36	43.7	35	0	4.1	1	4.14	6.1057	0.3072	54	87	33	37.7
A-37	44.1	35	0	4.5	1	4.14	6.1057	0.3072	60	88	28	32.4
A-38	36.8	35	0	3.1	1	4.14	6.1057	0.3072	41	61	20	32.8
A-39	36.1	35	0	3.2	1	4.14	6.1057	0.3072	42	74	32	42.8
A-40	28	35	0	8.6	1	4.14	6.1057	0.3072	114	104	10	9.4
A-41	32.1	35	0	6.3	1	4.14	6.1057	0.3072	83	105	22	20.6
A-42	28	35	0	6	1	4.14	6.1057	0.3072	79	90	11	11.8
A-43	31.8	35	0	6	1	4.14	6.1057	0.3072	79	97	18	18.2
A-44	32.7	35	0	6	1	4.14	6.1057	0.3072	79	92	13	13.7
A-45	34.3	35	0	5.6	1	4.14	6.1057	0.3072	74	81	7	8.6
A-46	27.7	35	0	8.8	1	4.14	6.1057	0.3072	116	127	11	8.4
A-47	32.4	35	0	7.1	1	4.14	6.1057	0.3072	94	121	27	22.4
A-48	31.7	35	0	9.5	1	4.14	6.1057	0.3072	126	165	39	23.9
A-49	31.3	35	0	11.6	1	4.14	6.1057	0.3072	153	152	1	0.9
A-50	28.4	35	0	4.49	1	4.14	6.1057	0.3072	59	113	54	47.4
A-51	29.5	35	0	5.9	1	4.14	6.1057	0.3072	78	97	19	19.6
A-52	34	35	0	5	1	4.14	6.1057	0.3072	66	102	36	35.2
A-53	35.6	35	0	5	1	4.14	6.1057	0.3072	66	107	41	38.2
A-54	17.3	35	0	6.2	1	4.14	6.1057	0.3072	82	115	33	28.7
A-55	18.7	35	0	9.1	1	4.14	6.1057	0.3072	120	115	5	4.7
A-56	17.7	35	0	7.8	1	4.14	6.1057	0.3072	103	71	32	45.3
A-57	20	35	0	4.3	1	4.14	6.1057	0.3072	57	72	15	21.0
A-58	17.9	35	0	6.1	1	4.14	6.1057	0.3072	81	78	3	3.4
A-59	13.9	35	0	11.6	1	4.14	6.1057	0.3072	153	202	49	24.1
A-60	13.7	35	0	14.8	1	4.14	6.1057	0.3072	196	213	17	8.1
A-61	17.7	35	0	7.3	1	4.14	6.1057	0.3072	97	124	27	22.1
A-62	11.7	35	0	12.1	1	4.14	6.1057	0.3072	160	128	32	25.0

A-63	16	35	0	9.8	1	4.14	6.1057	0.3072	130	97	33	33.6
A-64	17.1	35	0	8.6	1	4.14	6.1057	0.3072	114	90	24	26.4
A-65	16.6	35	0	8.8	1	4.14	6.1057	0.3072	116	108	8	7.8
A-66	21.1	35	0	5.3	1	4.14	6.1057	0.3072	70	102	32	31.3
A-67	21.3	35	0	8.3	1	4.14	6.1057	0.3072	110	107	3	2.6
A-68	21	35	0	9.9	1	4.14	6.1057	0.3072	131	117	14	11.9
A-69	10.1	35	0	7.5	1	4.14	6.1057	0.3072	99	145	46	31.6
A-70	16	35	0	7.7	1	4.14	6.1057	0.3072	102	142	40	28.3
A-71	9.7	35	0	10.2	1	4.14	6.1057	0.3072	135	143	8	5.7
A-72	14.6	35	0	9.8	1	4.14	6.1057	0.3072	130	148	18	12.4
A-73	20.6	35	0	9.7	1	4.14	6.1057	0.3072	128	141	13	9.0
A-74	25.4	25	0	7.4	1	4.14	5.2877	0.2822	82	141	59	42.0
A-75	24.1	50	0	9.2	1	4.14	7.3327	0.3447	154	181	27	14.9
A-76	24.7	75	0	9.5	1	4.14	9.3777	0.4072	222	232	10	4.1
A-77	13.9	25	0	13.7	1	4.14	5.2877	0.2822	151	186	35	18.6
A-78	13.7	50	0	15.9	1	4.14	7.3327	0.3447	266	284	18	6.2
A-79	13.7	75	0	16.9	1	4.14	9.3777	0.4072	396	333	63	18.8
A-80	46.3	75	1	8.1	1	4.14	9.3777	0.4072	135	122	13	11.0
A-81	47.7	75	0	9	1	4.14	9.3777	0.4072	211	176	35	19.7
A-82	8	75	0	12.4	1	4.14	9.3777	0.4072	290	190	100	52.8
A-83	18.5	75	0	11.3	1	4.14	9.3777	0.4072	265	167	98	58.4
A-84	31.7	75	0	6.8	1	4.14	9.3777	0.4072	159	139	20	14.5
A-85	10.8	75	0	12.5	1	4.14	9.3777	0.4072	293	245	48	19.5
A-86	22.2	75	0	9.9	1	4.14	9.3777	0.4072	232	226	6	2.6
A-87	33.1	75	0	8.3	1	4.14	9.3777	0.4072	194	168	26	15.7
A-88	22	75	0	9	1	4.14	9.3777	0.4072	211	163	48	29.3
A-89	24.7	35	1	7.4	1	4.14	6.1057	0.3072	70	125	55	44.1
A-90	25.7	50	1	8.4	1	4.14	7.3327	0.3447	101	142	41	29.2
A-91	27	75	1	7.4	1	4.14	9.3777	0.4072	124	178	54	30.5
A-92	14.5	25	1	13.4	1	4.14	5.2877	0.2822	106	111	5	4.7
A-93	14.7	35	1	16.7	1	4.14	6.1057	0.3072	158	165	7	4.4
A-94	13.7	50	1	15.8	1	4.14	7.3327	0.3447	189	211	22	10.4
A-95	13.6	75	1	14.1	1	4.14	9.3777	0.4072	236	238	2	0.9

A-96	10	35	1	8.9	1	4.14	6.1057	0.3072	84	104	20	19.2
A-97	27.6	35	1	10.8	1	4.14	6.1057	0.3072	102	115	13	11.3
A-98	28	35	0	11.5	1	4.14	6.1057	0.3072	152	149	3	2.1
A-99	27.3	75	0	11	1	4.14	9.3777	0.4072	258	219	39	17.6
A-100	30.9	75	1	10.4	1	4.14	9.3777	0.4072	174	189	15	8.0
Series B												
B-1	28.6	35	0	11.1	1	4.14	6.1057	0.3072	147	119	28	23.4
B-2	28.2	35	0	11.6	1	4.14	6.1057	0.3072	153	126	27	21.8
B-3	10.9	35	0	11.1	1	4.14	6.1057	0.3072	147	89	58	64.9
B-4	11	35	0	9.8	1	4.14	6.1057	0.3072	130	93	37	39.4
B-5	20.8	35	0	11.6	1	4.14	6.1057	0.3072	153	131	22	17.1
B-6	19	35	0	11.1	1	4.14	6.1057	0.3072	147	139	8	5.6
B-7	14.3	35	0	8.9	1	4.14	6.1057	0.3072	118	105	13	12.1
B-8	21	35	0	6.9	1	4.14	6.1057	0.3072	91	114	23	20.0
B-9	23.3	35	0	11.3	1	4.14	6.1057	0.3072	149	147	2	1.7
B-10	24.3	35	0	10.9	1	4.14	6.1057	0.3072	144	153	9	5.8
B-11	11	75	0	10	1	4.14	9.3777	0.4072	234	146	88	60.4
B-12	30	75	0	10.1	1	4.14	9.3777	0.4072	236	201	35	17.7
B-13	20.7	25	0	9.7	1	4.14	5.2877	0.2822	107	128	21	16.2
B-14	19.8	50	0	10.5	1	4.14	7.3327	0.3447	176	195	19	9.8
B-15	19.6	75	0	10	1	4.14	9.3777	0.4072	234	216	18	8.4
Series C												
C-1	136	35	0	9.36	0.4	4.14	6.1057	0.3072	93	108	15	13.5
C-2	136	35	1	9.36	0.4	4.14	6.1057	0.3072	48	88	40	45.3
C-3	70	35	0	6.75	1	4.14	6.1057	0.3072	89	145	56	38.4
C-4	70	35	1	6.75	1	4.14	6.1057	0.3072	64	90	26	29.2
C-5	175.7	35	0	6.75	0.4	4.14	6.1057	0.3072	67	87	20	22.6
C-6	174.6	35	1	6.75	0.4	4.14	6.1057	0.3072	48	52	4	7.5
Series D												
D-1	24.1	35	0	16.1	1	4.14	6.1057	0.3072	213	273	60	22.0

D-2	24.1	35	0	17.7	1	4.14	6.1057	0.3072	234	283	49	17.3
D-3	10	35	0	15.3	1	4.14	6.1057	0.3072	202	236	34	14.3
D-4	32.2	35	0	14.4	0.735	4.14	6.1057	0.3072	173	200	27	13.4
D-5	32	35	0	14.3	0.781	4.14	6.1057	0.3072	175	219	44	19.9
D-6	34	35	0	16.4	0.735	4.14	6.1057	0.3072	197	170	27	16.1
D-7	33	35	0	9.7	0.758	4.14	6.1057	0.3072	118	144	26	18.2
D-8	34	35	0	14.2	0.735	4.14	6.1057	0.3072	171	153	18	11.7
D-9	18	35	0	12.7	0.556	4.14	6.1057	0.3072	140	195	55	28.1
D-10	18	35	0	14.2	0.556	4.14	6.1057	0.3072	157	133	24	17.9
D-11	16	35	0	17.7	0.625	4.14	6.1057	0.3072	203	255	52	20.5
D-12	18	35	0	11	0.556	4.14	6.1057	0.3072	121	164	43	25.9
D-13	18	35	0	12.7	0.625	4.14	6.1057	0.3072	145	158	13	8.0
Series E												
E-1	15.5	35	0	11.1	1	4.14	6.1057	0.3072	147	174	27	15.6
E-2	16	35	0	13.6	1	4.14	6.1057	0.3072	180	133	47	35.2
E-3	14.3	35	0	14.1	1	4.14	6.1057	0.3072	186	196	10	4.9
E-4	22.7	35	0	5.3	1	4.14	6.1057	0.3072	70	81	11	13.5
E-5	25.7	35	0	14.586	1	4.14	6.1057	0.3072	193	135	58	42.9
E-6	23.7	35	0	10.4	1	4.14	6.1057	0.3072	138	138	0	0.3
E-7	22	35	0	12.5	1	4.14	6.1057	0.3072	165	187	22	11.6
E-8	22.1	35	0	13.6136	1	4.14	6.1057	0.3072	180	259	79	30.5
E-9	20.4	35	0	10.4	1	4.14	6.1057	0.3072	138	213	75	35.4
E-10	27.1	35	0	12.2	1	4.14	6.1057	0.3072	161	119	42	35.6
E-11	22.4	35	0	9.2	1	4.14	6.1057	0.3072	122	120	2	1.4
E-12	23.2	35	0	4.7	1	4.14	6.1057	0.3072	62	77	15	19.3
E-13	21.6	35	0	12.7	1	4.14	6.1057	0.3072	168	151	17	11.2
E-14	19.3	35	0	13.3	1	4.14	6.1057	0.3072	176	177	1	0.6
E-15	20.5	35	0	12.9064	1	4.14	6.1057	0.3072	171	172	1	0.8
E-16	23	35	0	5.8	1	4.14	6.1057	0.3072	77	88	11	12.8
E-17	20.3	35	0	6.188	1	4.14	6.1057	0.3072	82	107	25	23.5
Series F												

F-1	2.6	25	1	14.8	1	2.6	5.2877	0.2822	102	121	19	15.3
F-2	2.6	35	1	14.8	1	2.6	6.1057	0.3072	121	143	22	15.2
F-3	2.6	50	1	14.8	1	2.6	7.3327	0.3447	151	169	18	10.7
F-4	2.6	75	1	14.8	1	2.6	9.3777	0.4072	205	207	2	1.1
F-5	2.2	25	1	13.6	1	2.2	5.2877	0.2822	90	109	19	17.6
F-6	2.2	35	1	13.6	1	2.2	6.1057	0.3072	106	123	17	14.0
F-7	2.2	50	1	13.6	1	2.2	7.3327	0.3447	131	140	9	6.5
F-8	2.2	75	1	13.6	1	2.2	9.3777	0.4072	176	157	19	12.0
F-9	1.5	25	1	14.3	1	1.5	5.2877	0.2822	85	91	6	6.8
F-10	1.5	35	1	14.3	1	1.5	6.1057	0.3072	99	106	7	6.7
F-11	1.5	50	1	14.3	1	1.5	7.3327	0.3447	121	116	5	4.0
F-12	1.5	75	1	14.3	1	1.5	9.3777	0.4072	158	144	14	9.8
F-13	1.8	25	1	12.4	1	1.8	5.2877	0.2822	77	97	20	20.2
F-14	1.8	35	1	12.4	1	1.8	6.1057	0.3072	91	103	12	11.9
F-15	1.8	50	1	12.4	1	1.8	7.3327	0.3447	111	124	13	10.2
F-16	1.8	75	1	12.4	1	1.8	9.3777	0.4072	148	150	2	1.5
F-17	1.1	25	1	12.4	1	1.1	5.2877	0.2822	67	63	4	6.9
F-18	1.1	35	1	12.4	1	1.1	6.1057	0.3072	78	65	13	19.9
F-19	1.1	50	1	12.4	1	1.1	7.3327	0.3447	94	76	18	23.6
F-20	1.1	75	1	12.4	1	1.1	9.3777	0.4072	121	98	23	23.4
F-21	1.5	25	1	12.2	1	1.5	5.2877	0.2822	72	72	0	0.5
F-22	1.5	35	1	12.2	1	1.5	6.1057	0.3072	84	89	5	5.2
F-23	1.5	50	1	12.2	1	1.5	7.3327	0.3447	103	111	8	7.3
F-24	1.5	75	1	12.2	1	1.5	9.3777	0.4072	135	141	6	4.3
F-25	0.85	25	1	12.6	1	0.85	5.2877	0.2822	64	65	1	2.1
F-26	0.85	35	1	12.6	1	0.85	6.1057	0.3072	73	66	7	10.9
F-27	0.85	50	1	12.6	1	0.85	7.3327	0.3447	87	85	2	2.8
F-28	0.85	75	1	12.6	1	0.85	9.3777	0.4072	111	117	6	5.5
F-29	2.1	25	1	12.8	1	2.1	5.2877	0.2822	83	92	9	9.3
F-30	2.1	35	1	12.8	1	2.1	6.1057	0.3072	98	99	1	0.8
F-31	2.1	50	1	12.8	1	2.1	7.3327	0.3447	121	123	2	1.5
F-32	2.1	75	1	12.8	1	2.1	9.3777	0.4072	162	170	8	4.5
F-33	2.46	25	1	10.8	1	2.46	5.2877	0.2822	74	85	11	13.4

F-34	2.46	35	1	10.8	1	2.46	6.1057	0.3072	87	113	26	23.1
F-35	2.46	50	1	10.8	1	2.46	7.3327	0.3447	108	135	27	20.0
F-36	2.46	75	1	10.8	1	2.46	9.3777	0.4072	146	186	40	21.4
F-37	2.45	25	1	8.8	1	2.45	5.2877	0.2822	60	49	11	22.3
F-38	2.45	35	1	8.8	1	2.45	6.1057	0.3072	71	103	32	31.3
F-39	2.45	50	1	8.8	1	2.45	7.3327	0.3447	88	103	15	14.7
F-40	2.45	75	1	8.8	1	2.45	9.3777	0.4072	119	149	30	20.2
F-41	2.9	25	1	12.2	1	2.9	5.2877	0.2822	87	67	20	30.0
F-42	2.9	35	1	12.2	1	2.9	6.1057	0.3072	103	91	12	13.5
F-43	2.9	50	1	12.2	1	2.9	7.3327	0.3447	129	88	41	46.7
F-44	2.9	75	1	12.2	1	2.9	9.3777	0.4072	176	156	20	13.1
F-45	3.8	25	1	11.3	1	3.8	5.2877	0.2822	87	85	2	2.5
F-46	3.8	35	1	11.3	1	3.8	6.1057	0.3072	104	100	4	4.0
F-47	3.8	50	1	11.3	1	3.8	7.3327	0.3447	131	125	6	5.0
F-48	3.8	75	1	11.3	1	3.8	9.3777	0.4072	182	181	1	0.8
F-49	4.5	25	1	11.8	1	4.14	5.2877	0.2822	93	98	5	4.9
F-50	4.5	35	1	11.8	1	4.14	6.1057	0.3072	111	110	1	1.3
F-51	4.5	50	1	11.8	1	4.14	7.3327	0.3447	141	128	13	10.3
F-52	4.5	75	1	11.8	1	4.14	9.3777	0.4072	197	195	2	1.2
F-53	9.5	25	1	11.1	1	4.14	5.2877	0.2822	88	102	14	14.1
F-54	9.5	35	1	11.1	1	4.14	6.1057	0.3072	105	120	15	12.6
F-55	9.5	50	1	11.1	1	4.14	7.3327	0.3447	133	163	30	18.5
F-56	9.5	75	1	11.1	1	4.14	9.3777	0.4072	186	209	23	11.2
F-57	2.24	25	1	11.6	1	2.24	5.2877	0.2822	77	88	11	12.5
F-58	2.24	35	1	11.6	1	2.24	6.1057	0.3072	91	101	10	10.2
F-59	2.24	50	1	11.6	1	2.24	7.3327	0.3447	112	127	15	11.6
F-60	2.24	75	1	11.6	1	2.24	9.3777	0.4072	151	180	29	16.1
F-61	2.16	25	1	8.3	1	2.16	5.2877	0.2822	55	61	6	10.6
F-62	2.16	35	1	8.3	1	2.16	6.1057	0.3072	64	80	16	19.7
F-63	2.16	50	1	8.3	1	2.16	7.3327	0.3447	79	111	32	28.5
F-64	2.16	75	1	8.3	1	2.16	9.3777	0.4072	107	131	24	18.7
F-65	5.7	25	1	12.9	1	4.14	5.2877	0.2822	102	153	51	33.4
F-66	5.7	35	1	12.9	1	4.14	6.1057	0.3072	122	178	56	31.5

F-67	5.7	50	1	12.9	1	4.14	7.3327	0.3447	154	196	42	21.2
F-68	5.7	75	1	12.9	1	4.14	9.3777	0.4072	216	299	83	27.8
F-69	10.6	25	1	12.4	1	4.14	5.2877	0.2822	98	158	60	38.0
F-70	10.6	35	1	12.4	1	4.14	6.1057	0.3072	117	198	81	40.8
F-71	10.6	50	1	12.4	1	4.14	7.3327	0.3447	148	227	79	34.6
F-72	10.6	75	1	12.4	1	4.14	9.3777	0.4072	207	301	94	31.1
F-73	10.1	25	1	12.9	1	4.14	5.2877	0.2822	102	164	62	37.9
F-74	10.1	35	1	12.9	1	4.14	6.1057	0.3072	122	186	64	34.5
F-75	10.1	50	1	12.9	1	4.14	7.3327	0.3447	154	210	56	26.5
F-76	10.1	75	1	12.9	1	4.14	9.3777	0.4072	216	291	75	25.9
F-77	17.9	25	1	13.7	1	4.14	5.2877	0.2822	108	136	28	20.5
F-78	17.9	35	1	13.7	1	4.14	6.1057	0.3072	129	176	47	26.5
F-79	17.9	50	1	13.7	1	4.14	7.3327	0.3447	164	237	73	30.8
F-80	17.9	75	1	13.7	1	4.14	9.3777	0.4072	229	332	103	31.0
F-81	3.95	25	1	10.8	1	3.95	5.2877	0.2822	84	126	42	33.2
F-82	3.95	35	1	10.8	1	3.95	6.1057	0.3072	101	141	40	28.7
F-83	3.95	50	1	10.8	1	3.95	7.3327	0.3447	127	189	62	32.7
F-84	3.95	75	1	10.8	1	3.95	9.3777	0.4072	177	245	68	27.7
F-85	3.5	35	1	9.5	1	3.5	6.1057	0.3072	85	88	3	3.1
F-86	3.1	35	1	13.1	1	3.1	6.1057	0.3072	113	71	42	59.5
F-87	3.9	25	1	40	1	3.9	5.2877	0.2822	311	215	96	44.4
F-88	3.9	35	1	40	1	3.9	6.1057	0.3072	371	281	90	32.0
F-89	3.9	50	1	40	1	3.9	7.3327	0.3447	469	369	100	27.1
F-90	3.9	75	1	40	1	3.9	9.3777	0.4072	653	576	77	13.3
F-91	6.1	25	1	40	1	4.14	5.2877	0.2822	316	320	4	1.3
F-92	6.1	35	1	40	1	4.14	6.1057	0.3072	378	348	30	8.6
F-93	6.1	50	1	40	1	4.14	7.3327	0.3447	479	480	1	0.3
F-94	6.1	75	1	40	1	4.14	9.3777	0.4072	669	710	41	5.8
F-95	6.2	25	1	40	1	4.14	5.2877	0.2822	316	276	40	14.4
F-96	6.2	35	1	40	1	4.14	6.1057	0.3072	378	316	62	19.6
F-97	6.2	50	1	40	1	4.14	7.3327	0.3447	479	422	57	13.4
F-98	6.2	75	1	40	1	4.14	9.3777	0.4072	669	743	74	10.0
F-99	10.6	25	1	40	1	4.14	5.2877	0.2822	316	286	30	10.4

F-100	10.6	35	1	40	1	4.14	6.1057	0.3072	378	364	14	3.8
F-101	10.6	50	1	40	1	4.14	7.3327	0.3447	479	509	30	6.0
F-102	10.6	75	1	40	1	4.14	9.3777	0.4072	669	870	201	23.1
F-103	2	25	1	13.9	1	2	5.2877	0.2822	89	78	11	14.6
F-104	2	35	1	13.9	1	2	6.1057	0.3072	105	114	9	7.9
F-105	6.3	25	1	13.8	1	4.14	5.2877	0.2822	109	99	10	10.1
F-106	6.3	35	1	13.8	1	4.14	6.1057	0.3072	130	131	1	0.5
F-107	6.3	50	1	13.8	1	4.14	7.3327	0.3447	165	148	17	11.6
F-108	6.3	75	1	13.8	1	4.14	9.3777	0.4072	231	185	46	24.8
Series G												
G-1	3.7	35	1	17.9	1	3.7	6.1057	0.3072	163	192	29	14.9
G-2	2.7	35	1	19.7	1	2.7	6.1057	0.3072	163	150	13	8.8
G-3	3.5	35	1	22.8	1	3.5	6.1057	0.3072	205	236	31	13.3
G-4	1.4	35	1	15.2	1	1.4	6.1057	0.3072	103	107	4	3.8
G-5	2.3	35	1	14.9	1	2.3	6.1057	0.3072	118	131	13	10.3
G-6	2.2	35	1	18.8	0.9	2.2	6.1057	0.3072	142	176	34	19.6
G-7	3.8	50	1	17.5	1	3.8	7.3327	0.3447	203	255	52	20.3
G-8	2.7	50	1	18.3	1	2.7	7.3327	0.3447	189	222	33	14.9
G-9	3.5	50	1	23	1	3.5	7.3327	0.3447	260	321	61	19.1
G-10	1.5	50	1	15.9	1	1.5	7.3327	0.3447	134	143	9	6.2
G-11	2.3	50	1	14.9	1	2.3	7.3327	0.3447	146	174	28	16.3
G-12	2.2	50	1	20	0.9	2.2	7.3327	0.3447	186	249	63	25.5
Series H												
H-1	7.2	35	1	14.22	1	4.14	6.1057	0.3072	134	132	2	1.8
H-2	8.4	35	1	14.13	1	4.14	6.1057	0.3072	133	141	8	5.3
H-3	8.1	35	1	13.86	1	4.14	6.1057	0.3072	131	128	3	2.3
H-4	5.4	35	1	14.22	1	4.14	6.1057	0.3072	134	107	27	25.5
H-5	10.5	35	1	13.86	1	4.14	6.1057	0.3072	131	136	5	3.7
Series I												

I-1	24.8	75	0	3.3	1	4.14	9.3777	0.4072	77	43	34	79.7
I-2	8.4	35	0	6.8	1	4.14	6.1057	0.3072	90	80	10	12.4
I-3	8.6	50	0	6.8	1	4.14	7.3327	0.3447	114	80	34	42.4
I-4	8.9	75	0	6.4	1	4.14	9.3777	0.4072	150	170	20	11.9
I-5	10.9	35	0	0.3	1	4.14	6.1057	0.3072	4	8	4	50.4
I-6	11.6	50	0	1.5	1	4.14	7.3327	0.3447	25	17	8	47.8
I-7	10.85	75	0	3.1	1	4.14	9.3777	0.4072	73	26	47	179.2
I-8	2.8	25	0	27.3	1	2.8	5.2877	0.2822	270	279	9	3.1
I-9	2.8	35	0	28.6	1	2.8	6.1057	0.3072	335	297	38	12.9
I-10	2.8	50	0	28.2	1	2.8	7.3327	0.3447	413	355	58	16.3
I-11	4.8	50	0	25.6	1	4.14	7.3327	0.3447	429	276	153	55.4
I-12	4.8	50	0	27.8	1	4.14	7.3327	0.3447	466	357	109	30.5
I-13	4.8	50	0	28.6	1	4.14	7.3327	0.3447	479	381	98	25.8
I-14	14.4	50	0	4.2	1	4.14	7.3327	0.3447	70	73	3	3.6
I-15	17.9	50	0	5.7	1	4.14	7.3327	0.3447	95	88	7	8.5
Series J												
J-1	8.8	20	0	20.1178121	1	4.14	4.8787	0.2697	202	221	19	8.8
J-2	8.9	30	0	20.6746988	1	4.14	5.6967	0.2947	251	365	114	31.3
J-3	9	40	0	20.4285714	1	4.14	6.5147	0.3197	293	396	103	25.9
J-4	9.1	50	0	19.5775281	1	4.14	7.3327	0.3447	328	473	145	30.7
J-5	8.9	60	0	20.1882353	1	4.14	8.1507	0.3697	390	524	134	25.7
J-6	8.8	70	0	20.4321839	1	4.14	8.9687	0.3947	449	512	63	12.2
Series K												
K-1	9.9	75	0	15.5259259	1	4.14	9.3777	0.4072	364	275	89	32.2
K-2	11.6	50	0	13.2505747	1	4.14	7.3327	0.3447	222	222	0	0.0
K-3	12	50	0	12.8088889	1	4.14	7.3327	0.3447	215	227	12	5.5
K-4	11.5	50	0	13.3657971	1	4.14	7.3327	0.3447	224	218	6	2.7
K-5	9.5	35	0	16.1796491	1	4.14	6.1057	0.3072	214	195	19	9.7
K-6	10.2	35	0	15.069281	1	4.14	6.1057	0.3072	199	195	4	2.2
K-7	10.5	35	0	14.6387302	1	4.14	6.1057	0.3072	194	190	4	1.9
K-8	11.7	25	0	13.1373219	1	4.14	5.2877	0.2822	145	166	21	12.5

K-9	11	25	0	13.9733333	1	4.14	5.2877	0.2822	154	176	22	12.2
K-10	11.2	25	0	13.7238095	1	4.14	5.2877	0.2822	152	175	23	13.3
K-11	11.4	17	0	13.4830409	1	4.14	4.6333	0.2622	127	184	57	31.0
K-12	11	17	0	13.9733333	1	4.14	4.6333	0.2622	132	173	41	24.0
Series L												
L-1	3.4	35	0	32.04	1	3.4	6.1057	0.3072	399	355	44	12.4
L-2	6.8	35	0	32.04	1	4.14	6.1057	0.3072	424	403	21	5.1
L-3	5.1	25	0	32.04	1	4.14	5.2877	0.2822	354	335	19	5.7
L-4	10.2	25	0	32.04	1	4.14	5.2877	0.2822	354	340	14	4.2
L-5	5.1	35	0	32.04	1	4.14	6.1057	0.3072	424	355	69	19.4
L-6	10.2	35	0	32.04	1	4.14	6.1057	0.3072	424	380	44	11.5
L-7	5.1	45	0	32.04	1	4.14	6.9237	0.3322	498	370	128	34.6
L-8	10.2	45	0	32.04	1	4.14	6.9237	0.3322	498	405	93	22.9
Series M												
M-1	273	50	0	5.04	0.357	4.14	7.3327	0.3447	59	82	23	27.8
M-2	262	50	0	5.04	0.357	4.14	7.3327	0.3447	59	85	26	30.3
M-3	347.3	50	0	5.04	0.286	4.14	7.3327	0.3447	55	76.74	22	28.6
M-4	344.2	50	0	5.04	0.286	4.14	7.3327	0.3447	55	77.02	22	28.8
M-5	377	50	0	5.04	0.238	4.14	7.3327	0.3447	51	72.3	21	28.8
M-6	495.21	50	0	5.04	0.185	4.14	7.3327	0.3447	47	67.92	21	30.5
M-7	506	50	0	6.3	0.357	4.14	7.3327	0.3447	74	73.2	1	1.1
M-8	523	50	0	6.3	0.357	4.14	7.3327	0.3447	74	47.6	26	55.5
M-9	405.2	50	0	5.67	0.357	4.14	7.3327	0.3447	67	83.15	17	19.9
M-10	408.9	50	0	5.67	0.357	4.14	7.3327	0.3447	67	78.67	12	15.3
M-11	423	50	0	9.45	0.357	4.14	7.3327	0.3447	111	88.7	22	25.2
M-12	435	50	0	9.45	0.357	4.14	7.3327	0.3447	111	93.07	18	19.3
M-13	429	50	0	9.45	0.357	4.14	7.3327	0.3447	111	66.5	45	66.9
M-14	386	50	0	9.45	0.357	4.14	7.3327	0.3447	111	86	25	29.1
M-15	355.7	50	0	9.45	0.357	4.14	7.3327	0.3447	111	99.32	12	11.8
M-16	522	50	0	9.45	0.286	4.14	7.3327	0.3447	103	56.03	47	83.5
M-17	519.9	50	0	9.45	0.286	4.14	7.3327	0.3447	103	57.85	45	77.7

M-18	214.3	50	0	5.04	0.357	4.14	7.3327	0.3447	59	67.92	9	12.8
M-19	207.7	50	0	5.04	0.357	4.14	7.3327	0.3447	59	59.3	0	0.2
M-20	223	50	0	9.36	0.357	4.14	7.3327	0.3447	110	86	24	27.9
M-21	384	50	0	9.36	0.2	4.14	7.3327	0.3447	90	69	21	30.5
M-22	222	50	0	9.36	0.357	4.14	7.3327	0.3447	110	99	11	11.1
M-23	211	50	0	9.36	0.357	4.14	7.3327	0.3447	110	78	32	41.0
M-24	141	50	0	9.36	0.357	4.14	7.3327	0.3447	110	123	13	10.6
M-25	277	50	0	9.36	0.357	4.14	7.3327	0.3447	110	72	38	52.7
M-26	253	50	0	4.68	0.357	4.14	7.3327	0.3447	55	64	9	14.1
M-27	430	50	0	6.48	0.357	4.14	7.3327	0.3447	76	59	17	29.0
M-28	145	50	0	4.68	0.357	4.14	7.3327	0.3447	55	66	11	16.7
M-29	223	30	0	9.36	0.357	4.14	5.6967	0.2947	84	60	24	39.6
M-30	223	40	0	9.36	0.357	4.14	6.5147	0.3197	97	85	12	13.8
M-31	223	60	0	9.36	0.357	4.14	8.1507	0.3697	123	90	33	37.1
M-32	223	70	0	9.36	0.357	4.14	8.9687	0.3947	137	110	27	24.7
Series N												
N-1	1.2	25	0	15.6	1	1.2	5.2877	0.2822	122	110	12	10.5
N-2	1.2	35	0	15.6	1	1.2	6.1057	0.3072	141	124	17	13.7
N-3	1.2	50	0	15.6	1	1.2	7.3327	0.3447	171	136	35	25.4
N-4	1.2	75	0	15.6	1	1.2	9.3777	0.4072	221	200	21	10.3
N-5	140	35	0	12.2	1	4.14	6.1057	0.3072	161	185	24	12.8
N-6	140	50	0	12.2	1	4.14	7.3327	0.3447	204	198	6	3.2
N-7	140	75	0	12.2	1	4.14	9.3777	0.4072	286	235	51	21.6
N-8	220	35	0	10.2	1	4.14	6.1057	0.3072	135	165	30	18.2
N-9	220	50	0	10.2	1	4.14	7.3327	0.3447	171	176	5	2.9
N-10	220	75	0	10.2	1	4.14	9.3777	0.4072	239	283	44	15.6
N-11	82	35	0	13.5	1	4.14	6.1057	0.3072	179	159	20	12.3
N-12	85	35	0	11.5	1	4.14	6.1057	0.3072	152	163	11	6.7
N-13	98	35	0	13.3	1	4.14	6.1057	0.3072	176	164	12	7.3
N-14	200	35	0	13.5	1	4.14	6.1057	0.3072	179	160	19	11.6
N-15	200	50	0	13.5	1	4.14	7.3327	0.3447	226	241	15	6.2
N-16	200	75	0	13.5	1	4.14	9.3777	0.4072	316	320	4	1.2

N-17	215	35	0	13.8	1	4.14	6.1057	0.3072	183	161	22	13.4
N-18	215	50	0	13.8	1	4.14	7.3327	0.3447	231	197	34	17.4
N-19	215	75	0	13.8	1	4.14	9.3777	0.4072	323	251	72	28.7
N-20	175	35	0	9.6	1	4.14	6.1057	0.3072	127	136	9	6.6
N-21	175	50	0	9.6	1	4.14	7.3327	0.3447	161	153	8	5.1
N-22	165	50	0	11.6	1	4.14	7.3327	0.3447	194	198	4	1.9
N-23	165	75	0	11.6	1	4.14	9.3777	0.4072	272	244	28	11.3
N-24	230	35	0	12.4	1	4.14	6.1057	0.3072	164	212	48	22.6
N-25	230	50	0	12.4	1	4.14	7.3327	0.3447	208	177	31	17.4
N-26	143	50	0	12.6	1	4.14	7.3327	0.3447	211	181	30	16.6
N-27	250	50	0	12.4	1	4.14	7.3327	0.3447	208	208	0	0.1
N-28	100	35	0	13.6	1	4.14	6.1057	0.3072	180	185	5	2.8
N-29	100	50	0	13.6	1	4.14	7.3327	0.3447	228	207	21	10.1
N-30	100	75	0	13.6	1	4.14	9.3777	0.4072	318	266	52	19.7
Series O												
O-1	1	25	0	11.16	1	1	5.2877	0.2822	83	111	28	25.6
O-2	1	35	0	11.16	1	1	6.1057	0.3072	95	124	29	23.1
O-3	1	50	0	11.16	1	1	7.3327	0.3447	115	136	21	15.8
O-4	1	75	0	11.16	1	1	9.3777	0.4072	147	200	53	26.7
O-5	12.5	25	0	11.16	1	4.14	5.2877	0.2822	123	126	3	2.1
O-6	12.5	35	0	11.16	1	4.14	6.1057	0.3072	148	145	3	1.8
O-7	12.5	50	0	11.16	1	4.14	7.3327	0.3447	187	186	1	0.5
O-8	12.5	75	0	11.16	1	4.14	9.3777	0.4072	261	220	41	18.8
O-9	6	25	0	12.06	1	4.14	5.2877	0.2822	133	230	97	42.0
O-10	6	35	0	12.06	1	4.14	6.1057	0.3072	159	266	107	40.0
O-11	6	50	0	12.06	1	4.14	7.3327	0.3447	202	272	70	25.7
O-12	6	75	0	12.06	1	4.14	9.3777	0.4072	282	372	90	24.1
O-13	8	25	0	12.06	1	4.14	5.2877	0.2822	133	145	12	8.1
O-14	8	35	0	12.06	1	4.14	6.1057	0.3072	159	153	6	4.2
O-15	8	50	0	12.06	1	4.14	7.3327	0.3447	202	178	24	13.5
O-16	8	75	0	12.06	1	4.14	9.3777	0.4072	282	229	53	23.3
O-17	29	50	0	12.06	1	4.14	7.3327	0.3447	202	191	11	5.8

O-18	100	35	0	12.24	1	4.14	6.1057	0.3072	162	157	5	3.1
O-19	53	50	0	12.24	1	4.14	7.3327	0.3447	205	180	25	13.9
O-20	110	35	0	11.52	1	4.14	6.1057	0.3072	152	172	20	11.4
O-21	56	50	0	11.52	1	4.14	7.3327	0.3447	193	207	14	6.8
O-22	150	35	0	11.43	1	4.14	6.1057	0.3072	151	161	10	6.1
O-23	67	50	0	11.43	1	4.14	7.3327	0.3447	191	198	7	3.3
O-24	110	35	0	12.42	1	4.14	6.1057	0.3072	164	174	10	5.6
O-25	50	35	0	12.42	1	4.14	6.1057	0.3072	164	193	29	14.9
O-26	50	50	0	12.42	1	4.14	7.3327	0.3447	208	166	42	25.3
O-27	50	75	0	12.42	1	4.14	9.3777	0.4072	291	243	48	19.7
O-28	102	50	0	12.42	1	4.14	7.3327	0.3447	208	178	30	16.9
O-29	33	50	0	12.42	1	4.14	7.3327	0.3447	208	187	21	11.3
O-30	110	35	0	12.06	1	4.14	6.1057	0.3072	159	165	6	3.3
O-31	71	35	0	12.06	1	4.14	6.1057	0.3072	159	189	30	15.6
O-32	71	50	0	12.06	1	4.14	7.3327	0.3447	202	197	5	2.6
O-33	71	75	0	12.06	1	4.14	9.3777	0.4072	282	273	9	3.4
O-34	117	50	0	12.06	1	4.14	7.3327	0.3447	202	228	26	11.4
O-35	44	50	0	12.06	1	4.14	7.3327	0.3447	202	208	6	2.9
O-36	95	35	0	12.15	1	4.14	6.1057	0.3072	161	154	7	4.3
O-37	51	50	0	12.15	1	4.14	7.3327	0.3447	204	213	9	4.4
Series P												
P-1	45.6122	35	0	11.7	1	4.14	6.1057	0.3072	155	120	35	28.9
P-2	47.8571	35	0	11.3	1	4.14	6.1057	0.3072	149	134	15	11.5
P-3	58.6735	35	0	12.65	1	4.14	6.1057	0.3072	167	126.5	41	32.3
P-4	63.6735	35	0	12.45	1	4.14	6.1057	0.3072	165	128.5	36	28.1
P-5	69.5918	35	0	9.1	1	4.14	6.1057	0.3072	120	145	25	17.0
P-6	111.837	35	0	14.4	1	4.14	6.1057	0.3072	190	143.5	47	32.7
P-7	125.714	35	0	13.9	1	4.14	6.1057	0.3072	184	199	15	7.6
Series Q												
Q-1	75.3	50	0	10.8	1	4.14	7.3327	0.3447	181	201	20	10.0
Q-2	55.8	50	0	11.7	1	4.14	7.3327	0.3447	196	187	9	4.8

Q-3	64.4	50	0	10.89	1	4.14	7.3327	0.3447	182	229	47	20.3
Q-4	62.9	50	0	11.88	1	4.14	7.3327	0.3447	199	193	6	3.1
Q-5	54.7	50	0	12.78	1	4.14	7.3327	0.3447	214	274	60	21.9
Q-6	82.5	50	0	9.72	1	4.14	7.3327	0.3447	163	219	56	25.6
Q-7	65.1	50	0	11.07	1	4.14	7.3327	0.3447	185	223	38	16.8
